# Trends in the global burden of vision loss among the older adults from 1990 to 2019

**DOI:** 10.3389/fpubh.2024.1324141

**Published:** 2024-04-04

**Authors:** Jiayang Yin, Bing Jiang, Tantai Zhao, Xiaojian Guo, Yao Tan, Yanbing Wang

**Affiliations:** ^1^Department of Ophthalmology, The Third Xiangya Hospital, Central South University, Changsha, Hunan, China; ^2^Postdoctoral Station of Clinical Medicine, The Third Xiangya Hospital, Central South University, Changsha, Hunan, China; ^3^Department of Ophthalmology, The Second Xiangya Hospital, Central South University, Changsha, Hunan, China

**Keywords:** older adults, vision loss, average annual percentage changes, sociodemographic index, years lived with disability

## Abstract

**Purpose:**

To quantify the global impact of vision impairment in individuals aged 65 years and older between 1990 and 2019, segmented by disease, age, and sociodemographic index (SDI).

**Methods:**

Using the Global Burden of Diseases 2019 (GBD 2019) dataset, a retrospective demographic evaluation was undertaken to ascertain the magnitude of vision loss over this period. Metrics evaluated included case numbers, prevalence rates per 100,000 individuals, and shifts in prevalence rates via average annual percentage changes (AAPCs) and years lived with disability (YLDs).

**Results:**

From 1990 to 2019, vision impairment rates for individuals aged 65 years and older increased from 40,027.0 (95% UI: 32,232.9-49,945.1) to 40,965.8 (95% UI: 32,911-51,358.3, AAPC: 0.11). YLDs associated with vision loss saw a significant decrease, moving from 1713.5 (95% UI: 1216.2–2339.7) to 1579.1 (95% UI: 1108.3–2168.9, AAPC: −0.12). Gender-based evaluation showed males had lower global prevalence and YLD rates compared to females. Cataracts and near vision impairment were the major factors, raising prevalence by 6.95 and 2.11%, respectively. Cataract prevalence in high-middle SDI regions and near vision deficits in high SDI regions significantly influenced YLDs variation between 1990 and 2019.

**Conclusion:**

Over the past three decades, there has been a significant decrease in the vision impairment burden in individuals aged 65 and older worldwide. However, disparities continue, based on disease type, regional SDI, and age brackets. Enhancing eye care services, both in scope and quality, is crucial for reducing the global vision impairment burden among the older adults.

## 1 Introduction

The global population has experienced a rapid aging trend in recent decades, largely due to socioeconomic development. This demographic shift has resulted in an increasing burden of disease on healthcare systems worldwide, particularly regarding certain types of diseases and injuries. According to World Population Prospects 2019 (1), the number of people aged 65 years and older is projected to rise from 1 in 11 people worldwide in 2019 to 1 in 6 people by 2050. Furthermore, population aging has been associated with a significant increase in global disability-adjusted life years (2), highlighting the need for increased medical resources to meet the healthcare needs of the older adults. As a result, many healthcare systems will require reforms to cope with the health impact of population aging.

While previous studies have examined specific aspects of the health impact of population aging (3, 4), few have explored the relationship between population aging and vision loss on a global scale. Some studies have focused on specific regions (5–8) or diseases (9–11), making it difficult to draw generalized conclusions. Additionally, some studies have not separated the effects of population aging from population growth (12, 13), leading to inaccurate estimates of the net effect of population aging. Furthermore, traditional decomposition methods used in previous studies are sensitive to decomposition order and choice of reference group, leading to inconsistent results (3, 14).

To address these limitations, we present a systematic analysis of the health impact of global population aging and vision loss between 1990 and 2019. We utilized a decomposition method that is not influenced by decomposition order or choice of reference group, allowing for accurate estimation of the net effect of population aging on vision loss (3). Our study aims to provide valuable information for policymakers and researchers to better understand and address the healthcare needs of the aging population, especially regarding vision loss.

## 2 Methods

### 2.1 Overview

The data source on population aging and vision loss during 1990–2019 were acquired from the GBD 2019 (15), which provided estimates of health outcomes and related measures for countries and territories worldwide. The data included prevalence of vision loss by sex, age group, and country from 1990 to 2019. In this study, the definition and diagnosis of vision loss adhered strictly to the criteria set forth by the GBD 2019 (16). Specifically, our classification utilized the Snellen chart standards to define moderate vision impairment as presenting visual acuity (PVA) of greater than or equal to 6/60 and less than 6/18, severe vision impairment as PVA of greater than or equal to 3/60 and less than 6/60, and blindness as PVA less than 3/60 or a visual field around central fixation of less than 10 degrees. GBD 2019 used multiple data sources, including surveys, censuses, and administrative records to estimate the prevalence of vision loss. The study classified the countries and territories into high-income, upper-middle-income, lower-middle-income, and low-income categories based on the World Bank’s income classifications in 2019.

### 2.2 Frontier analysis method

To assess the correlation between the burden of vision loss and socio-demographic development, we utilized a quantitative methodology known as frontier analysis. This approach aimed to identify the lowest achievable age-standardized YLDs rate based on the Socio-demographic Index (SDI), which serves as a measure of development status. The YLDs frontier represents the minimum YLDs that each country or territory could potentially attain given its specific SDI value. The effective difference, which measures the distance from the frontier, indicates the extent to which there may be unrealized opportunities for improvement (reduction in YLDs) based on a country or territory’s position on the development spectrum. To construct the frontier for age-adjusted vision loss YLDs by SDI, we employed a data envelope analysis method called the free disposal hull, which allows for non-linear frontiers (17, 18). Data from 1990 to 2019 were utilized, and to address uncertainty, we generated 1,000 bootstrapped samples by randomly selecting data points with replacement from all countries and territories across the years. The mean YLDs attributed to vision loss was calculated for each Socio-demographic Index (SDI) value based on the bootstrapped samples. Subsequently, a smoothed frontier was generated using LOESS regression with a local polynomial degree of 1 and a span of 0.2. Outliers were excluded during the frontier generation process to mitigate their influence (17). To examine the relationship between age-standardized vision loss YLDs rates and the frontier in 2019, we calculated the effective difference, which represents the absolute distance from the frontier, using the 2019 SDI and age-standardized vision loss YLDs rate data point for each country or territory. Countries or territories with lower YLDs than the frontier were assigned a zero distance, indicating that they had achieved or surpassed the minimum YLDs level established by their SDI value.

### 2.3 Decomposition method

In our study, we sought to analyze the shifts in vision loss prevalence from 1990 to 2019 by attributing these changes to three primary factors: population aging, population growth, and variations in age-specific prevalence rates. To accomplish this, we embarked on a detailed comparative analysis of several decomposition methods that have been documented in existing research (3, 4, 14, 19, 20). These methods, each offering unique insights into the breakdown of health outcome changes, often face common challenges, notably in the selection of decomposition order and reference groups, leading to possible inconsistencies in outcomes.

After an exhaustive evaluation and comparison of these methodologies, we adopted a specific decomposition approach that effectively addresses the noted challenges, ensuring a consistent and clear framework for attributing changes in vision loss prevalence (14). This selected method employs a comprehensive set of formulas to systematically calculate the impact of population aging, growth, and changes in mortality rates on vision loss variation. It stands out for its methodological robustness and consistency, making it particularly suitable for our analysis. For our data analysis, we utilized R version 4.2.3, leveraging the “maps” package to create detailed visualizations that complement our findings. For our data analysis, we utilized R version 4.2.3, leveraging the “maps” package to create detailed visualizations that complement our findings. To conduct the decomposition analysis, we employed the easyGBDR package, version 1.0.0.1.

Using the aforementioned decomposition method, we computed the absolute and relative contributions of population aging, population growth, and changes in age-specific prevalence rates to the disparity in vision loss prevalence between 1990 and each subsequent year from 1991 to 2019. These calculations encompassed the global population as well as individual countries and territories considered in our study. The absolute contribution represents the number of vision loss cases attributed to each factor, while the relative contribution (“attributed proportion”) is expressed as a percentage, representing the attributed cases of vision loss divided by the total number of cases in 1990. A positive contribution indicates an increase in the prevalence of vision loss, while a negative contribution indicates a decrease.

### 2.4 Statistical analysis

We employed visualizations to illustrate the absolute contributions of the three components to changes in vision loss prevalence. Additionally, we graphically depicted the relative contributions of population aging by sex for the global population and various income categories as classified by the World Bank. Moreover, we compiled tables showcasing the top five causes of vision loss (including glaucoma, cataracts, AMD, refractive disorders, and near vision loss) with the most substantial increases and decreases in attributed cases related to population aging, categorized by sex. Furthermore, we estimated the relative contributions of population aging from 1990 to 2019, stratified by country, sex, and specific causes of vision loss.

To further deepen our understanding of the interplay between population aging and changes in vision loss prevalence, we calculated the ratio of vision loss cases attributed to changes in age-specific prevalence rates to those attributed to population aging. This analysis focused on countries where population aging corresponded to an increase in vision loss prevalence between 1990 and 2019. By utilizing this ratio, we assessed the comparative impact of changes in age-specific prevalence rates versus population aging on alterations in vision loss prevalence. Notably, all analyses were stratified by sex, recognizing the divergent effects of population aging on vision loss prevalence between males and females.

Our analysis strategy was finalized in March 2023, and involved exploring patterns in the prevalence of vision loss attributed to population aging, variation in the number of attributed cases, and changes in the number of attributed cases by sex, country income category, and cause of vision loss. We also compared the effect of changes in age-specific prevalence rates to the effect of population aging. All data analyses were conducted between April 2023 and May 2023. We followed the Guidelines for Accurate and Transparent Health Estimates Reporting (GATHER) statement (21) to ensure transparency and accuracy in our research.

## 3 Results

### 3.1 Overview of the global burden

**Figure 1:**
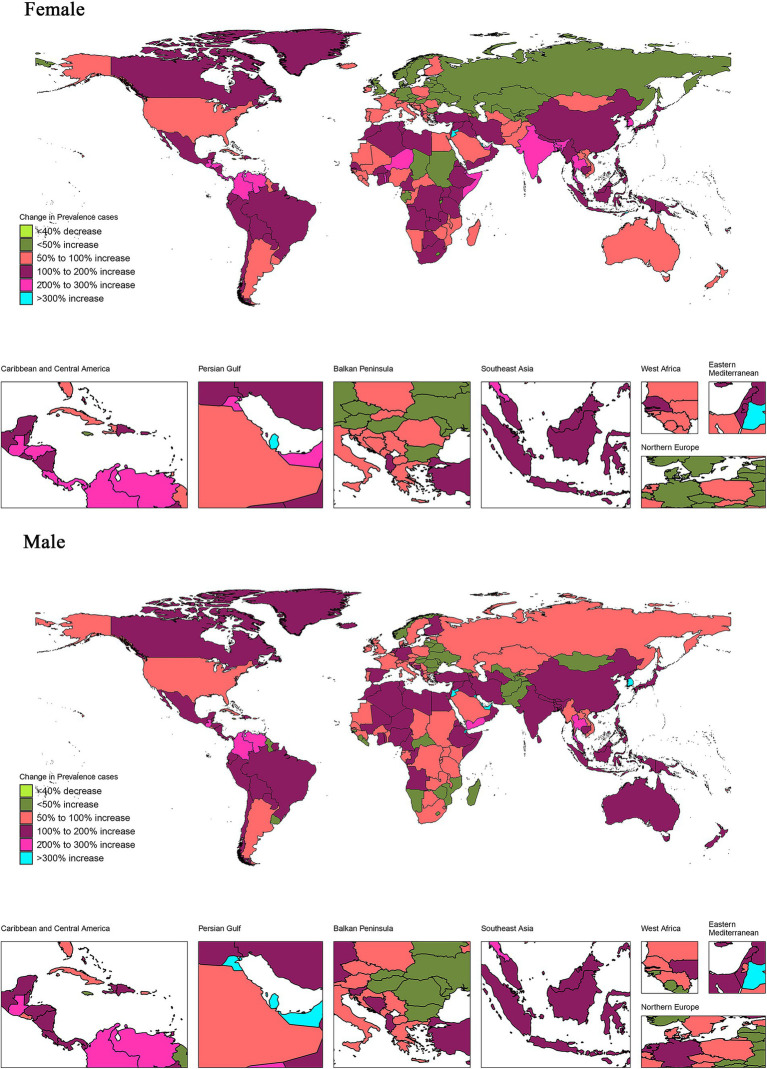
Proportion of changes in prevalence number associated with vision loss between 1990 and 2019 in 204 countries and regions. The proportion of change in prevalence number was calculated as the change in prevalence number between 1990 and 2019 divided by prevalence number in 1990 × 100%. Countries and regions with negative proportions were treated as a single category. Countries and regions with positive proportions were classified into 5 categories according to quintiles of positive proportions. The maps were drawn using the R package “maps,” which was based on the data from the Natural Earth project.

**Figure 2:**
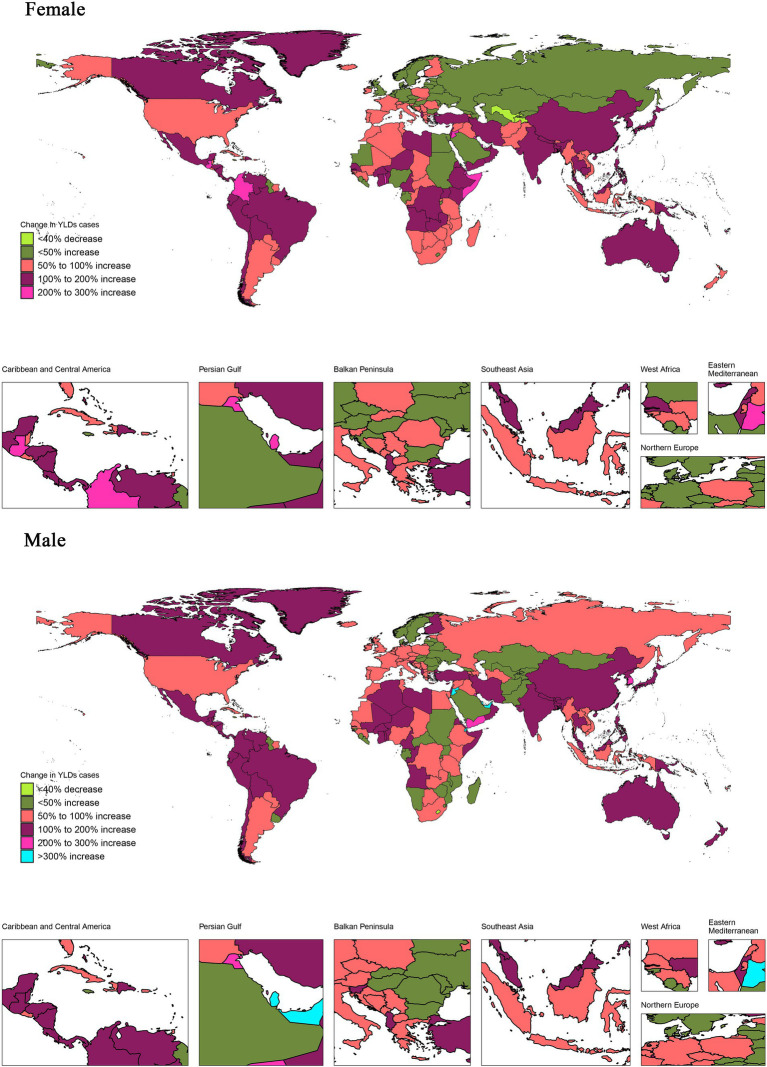
Proportion of changes in YLDs associated with vision loss between 1990 and 2019 in 204 countries and regions. The proportion of change in YLDs was calculated as the change in YLD between 1990 and 2019 divided by YLD in 1990 × 100%. Countries and regions were categorized into a single group with negative proportions, while those with positive proportions were divided into five categories based on quintiles. YLDs, years lived with disability.

Globally, there has been a significant upward trend in the prevalence and AAPC of global vision loss (Figure 1 and Table 1). The age-standardized prevalence rate (ASPR) increased from 40,027.0 (95% uncertainty interval [UI] = 32,232.9–49,945.1) in 1990 to 40,965.8 (95% UI = 32,911–51,358.3) in 2019. The AAPC for prevalence showed an increase of 0.11 (95% confidence interval [CI]: 0.07 to 0.14). Conversely, the number of YLDs and its average annual percentage change exhibited a declining trend, with an AAPC of −0.12 (95% CI: −0.23 to −0.01) (Figure 2 and Table 1). Overall, the YLDs of vision loss decreased for all age groups (age ≥ 65 years) and SDI groups from 1990 to 2019. In terms of vision loss due to specific eye diseases, age-standardized prevalence rate per 100,000 population increased for vision loss due to cataract and near vision loss; it decreased for vision loss due to glaucoma, age-related macular degeneration (AMD), refraction disorders and other causes (Table 2). YLD rate per 100,000 population increased during the monitoring period for near vision loss; it decreased for glaucoma, cataract, AMD, refraction disorders and other causes (Table 3). Notably, the high SDI group showed a significant upward trend in the age-standardized rate and YLD rate of near vision loss from 1990 to 2019, while the other SDI groups showed significant decreases in both rates.

**Table 1:** Prevalence and years lived with disability (YLDs) for overall vision loss in global and regional populations (age ≥ 65 years) from 1990 to 2019.

	Prevalence						YLDs					
	1990		2019		AAPC,1990–2019	*p*-value	1990		2019		AAPC,1990–2019	*p*-value
	cases (n)	ASPR (per 100,000 population)	cases (n)	ASPR (per 100,000 population)			cases (n)	ASYR (per 100,000 population)	cases (n)	ASYR (per 100,000 population)		
Global	127,849,966	40027.0 (32232.9–49945.1)	293,674,327	40965.8 (32911–51358.3)	0.11 (0.07 to 0.14)	<0.001	5,322,245	1713.5 (1216.2–2339.7)	11,187,620	1579.1 (1108.3–2168.9)	−0.12 (−0.23 to −0.01)	**0.035**
Sex												
Male	53,164,877	39287.2 (31570.5–49178.7)	125,601,695	39027.5 (31281.6–49128.1)	0.01 (−0.06 to 0.08)	0.734	2,185,399	1691.6 (1202.3–2,309)	4,677,036	1492.2 (1045.1–2053.1)	−0.28 (−0.38 to −0.18)	**<0.001**
Female	74,685,089	40606.3 (32681.8–50553.7)	168,072,632	42567.6 (34208.9–53315.5)	0.19 (0.17 to 0.21)	<0.001	3,136,846	1733.9 (1232.2–2369.7)	6,510,584	1,651 (1160.4–2266.8)	−0.05 (−0.16 to 0.07)	0.43
Age group, years												
65–69	42,101,113	34093.1 (26388.3–43397.7)	92,267,021	35681.7 (27642–45,366)	0.15 (0.09 to 0.2)	<0.001	1,492,096	1208.3 (839.3–1696.6)	2,926,040	1131.6 (776.1–1603.7)	−0.2 (−0.31 to −0.09)	**<0.001**
70–74	33,638,614	39802.7 (32000.2–49428.2)	74,687,619	39921.3 (32083–49895.1)	0.03 (−0.02 to 0.07)	0.23	1,363,851	1613.8 (1151.3–2210.4)	2,719,515	1453.6 (1017.5–2000.2)	−0.25 (−0.39 to −0.11)	**0.001**
75–79	26,224,165	42773.6 (34876.7–53931.8)	55,748,941	43878.2 (35758.5–55360.9)	0.09 (0 to 0.18)	0.042	1,148,318	1873.0 (1341.1–2562.5)	2,259,834	1778.6 (1262.7–2443.9)	−0.15 (−0.24 to −0.06)	**0.001**
80–84	16,073,570	45638.1 (37659.8–56516.7)	39,704,725	47031.1 (38616.8–58925.7)	0.17 (0 to 0.35)	0.055	775,072	2200.7 (1569.6–2,952)	1,728,723	2047.7 (1450.5–2758.2)	−0.06 (−0.36 to 0.25)	0.706
85–89	7,175,646	47619.5 (39848.7–57464.6)	20,851,596	47955.8 (39776–58380.2)	0.04 (−0.04 to 0.12)	0.294	382,115	2535.8 (1821.1–3366.8)	993,074	2283.9 (1629.1–3039.1)	−0.24 (−0.46 to −0.02)	**0.029**
90–94	2,124,299	48213.7 (40977.2–56546.7)	8,135,642	48261.0 (40534.5–57555.6)	−0.01 (−0.06 to 0.05)	0.784	126,645	2874.4 (2049–3778.9)	426,615	2530.7 (1795.4–3350.5)	−0.42 (−0.5 to −0.34)	**<0.001**
95+	512,559	49789.8 (41592.1–58369.7)	2,278,781	47741.1 (39223.2–57022.1)	−0.14 (−0.18 to −0.09)	<0.001	34,145	3316.8 (2336.7–4428.6)	133,820	2803.6 (1983.4–3792.5)	−0.55 (−0.58 to −0.52)	**<0.001**
Sociodemographic index												
High	13,179,895	13451.5 (10971.5–16493.9)	25,520,118	13412.5 (10898.5–16543.5)	0.04 (0 to 0.09)	0.069	535,161	550.0 (384–754.2)	983,828	507.9 (352.6–700.4)	−0.15 (−0.22 to −0.09)	**<0.001**
High-middle	37,221,322	42877.7 (33641.2–54823.5)	77,614,558	42228.3 (33190.1–53974.5)	−0.03 (−0.06 to 0)	0.09	1,225,703	1465.5 (1016.5–2035.6)	2,459,535	1353.7 (929.3–1897.8)	−0.07 (−0.15 to 0.02)	0.118
Middle	39,984,412	54459.6 (43650.1–68235.1)	102,118,249	51049.5 (40809.1–64436.2)	−0.19 (−0.24 to −0.13)	<0.001	1,745,223	2524.4 (1788.8–3429.6)	3,959,525	2046 (1440.9–2804.7)	−0.51 (−0.76 to −0.25)	**<0.001**
Low-middle	26,830,514	63909.8 (52799.9–77,796)	65,087,888	59989.6 (48880.1–74110.3)	−0.18 (−0.21 to −0.14)	<0.001	1,347,089	3391.7 (2427.6–4574.6)	2,818,977	2688.2 (1900.9–3671.3)	−0.66 (−0.77 to −0.54)	**<0.001**
Low	10,570,892	64551.3 (52595.5–78745.1)	23,202,944	62630.6 (51024.7–76932.4)	−0.08 (−0.16 to −0.01)	0.032	466,193	3055.8 (2183.3–4134.7)	960,346	2717.8 (1928.6–3,714)	−0.29 (−0.42 to −0.16)	**<0.001**
Region												
Andean Latin America	762,685	48675.6 (39871.5–60332.6)	2,164,611	45651.5 (36650.4–57073.9)	−0.25 (−0.32 to −0.18)	<0.001	762,685	2535.4 (1775.6–3490.8)	2,164,611	1934.8 (1337.1–2,673)	−0.88 (−1.08 to −0.68)	**<0.001**
Australasia	265,990	12422.6 (10523.7–14595.4)	558,935	11331.1 (9215.5–13,898)	−0.25 (−0.4 to −0.1)	0.001	265,990	511.8 (355.6–698.3)	558,935	472.6 (328.2–645.3)	−0.1 (−0.26 to 0.07)	0.238
Caribbean	957,101	42820.3 (33227.6–55719.4)	1,833,112	39842.1 (30782.5–51781.5)	−0.21 (−0.23 to −0.19)	<0.001	957,101	1649.8 (1142.7–2293.5)	1,833,112	1362.4 (932.9–1903.1)	−0.59 (−0.61 to −0.56)	**<0.001**
Central Asia	1,900,830	55285.9 (43144.1–70434.6)	2,545,400	53161.6 (41152–68593.7)	−0.14 (−0.2 to −0.08)	<0.001	1,900,830	1946.3 (1336.9–2727.4)	2,545,400	1697.3 (1153–2,393)	−0.51 (−0.62 to −0.4)	**<0.001**
Central Europe	5,909,295	46852.7 (34413–63,050)	9,458,374	44670.3 (32795.2–60137.9)	−0.06 (−0.1 to −0.01)	0.014	5,909,295	1022.4 (654.2–1549.7)	9,458,374	938.3 (590.7–1,439)	−0.16 (−0.23 to −0.09)	**<0.001**
Central Latin America	3,117,808	49724.8 (39478.6–63094.1)	9,387,270	47618.2 (37079.5–61068.8)	−0.11 (−0.14 to −0.08)	<0.001	3,117,808	2,158 (1515.4–2954.8)	9,387,270	1702 (1178.1–2359.8)	−0.72 (−0.85 to −0.58)	**<0.001**
Central Sub-Saharan Africa	834,794	57028.3 (42104.4–75112.5)	1,803,244	54942.6 (40808.1–73074.3)	−0.1 (−0.24 to 0.04)	0.158	834,794	1481.8 (983.6–2159.1)	1,803,244	1379.3 (906.4–2031)	−0.07 (−0.25 to 0.11)	0.462
East Asia	34,284,300	52953.3 (40390.4–69197.6)	89,049,826	49475.8 (38057.9–64,462)	−0.17 (−0.27 to −0.07)	0.001	34,284,300	1694.8 (1166.3–2375.1)	89,049,826	1486.1 (1002.6–2103.4)	−0.19 (−0.78 to 0.41)	0.536
Eastern Europe	12,730,806	55123.3 (42491.1–70967.3)	18,022,574	56232.3 (43032.7–72855.4)	0.06 (0.02 to 0.11)	0.003	12,730,806	1574.1 (1056.4–2265.4)	18,022,574	1,460 (960–2154.9)	−0.19 (−0.28 to −0.11)	**<0.001**
Eastern Sub-Saharan Africa	3,364,901	64,271 (50586.8–80,276)	6,654,041	60,133 (47150.5–76049.8)	−0.22 (−0.27 to −0.17)	<0.001	3,364,901	2738.5 (1934.7–3727.8)	6,654,041	2388.6 (1687.5–3277.7)	−0.45 (−0.58 to −0.32)	**<0.001**
High-income Asia Pacific	1,592,137	9667.9 (8015.8–11621.9)	4,687,725	9480.7 (7816–11449.3)	0.2 (0.12 to 0.28)	<0.001	1,592,137	484.2 (338.7–660.4)	4,687,725	450.3 (312.2–618.5)	0.07 (−0.12 to 0.25)	0.482
High-income North America	3,748,514	10868.7 (8784.5–13500.7)	6,571,011	10716.5 (8651–13330.8)	−0.05 (−0.12 to 0.02)	0.178	3,748,514	427.3 (300.5–582.1)	6,571,011	406.6 (285.5–554.9)	−0.17 (−0.26 to −0.07)	**0.001**
North Africa and Middle East	5,400,635	44883.5 (37856.3–53947.6)	12,279,852	39775.1 (33147.4–48189.7)	−0.34 (−0.41 to −0.28)	<0.001	5,400,635	2747.3 (1943.8–3730.4)	12,279,852	2018.6 (1421.8–2744.5)	−0.96 (−1.05 to −0.86)	**<0.001**
Oceania	104,332	55621.9 (45526.5–68218.6)	231,532	54142.2 (44062.8–66668.9)	−0.07 (−0.23 to 0.09)	0.413	104,332	2327.4 (1619.3–3205.1)	231,532	2115.5 (1466.1–2924.9)	−0.29 (−0.58 to 0.01)	0.061
South Asia	27,196,147	71820.5 (60767.3–85211.9)	74,805,014	66778.6 (55349.7–81070.5)	−0.21 (−0.24 to −0.18)	<0.001	27,196,147	4087.6 (2937.6–5478.9)	74,805,014	3143.5 (2236.8–4268.4)	−0.76 (−0.88 to −0.63)	**<0.001**
Southeast Asia	9,833,193	55732.9 (47128.6–66380.4)	22,442,888	50644.9 (42552.2–61298.2)	−0.31 (−0.37 to −0.24)	<0.001	9,833,193	3607.5 (2573.5–4844.8)	22,442,888	2680.3 (1908.7–3,614)	−1 (−1.12 to −0.88)	**<0.001**
Southern Latin America	592,537	15296.7 (12836.4–18084.1)	1,125,434	14479.5 (12086.1–17191.8)	−0.03 (−0.08 to 0.01)	0.125	592,537	816.8 (568.5–1118.1)	1,125,434	708.3 (489.9–974.5)	−0.27 (−0.33 to −0.22)	**<0.001**
Southern Sub-Saharan Africa	1,540,857	71661.6 (54521.1–89290.9)	2,939,557	67931.4 (51008.7–87105.2)	−0.18 (−0.2 to −0.15)	<0.001	1,540,857	2453.2 (1704.7–3,392)	2,939,557	2016.4 (1390.2–2845.9)	−0.7 (−0.85 to −0.54)	**<0.001**
Tropical Latin America	2,838,342	42423.8 (34591.6–52836.7)	7,955,527	39029.6 (31453.8–49169.8)	−0.12 (−0.31 to 0.06)	0.19	2,838,342	2135.3 (1514.9–2886.4)	7,955,527	1745.5 (1230.4–2383.6)	−0.51 (−0.79 to −0.23)	**<0.001**
Western Europe	7,066,664	12659.2 (10610.2–15053.4)	11,553,114	12050.1 (10056.4–14401.6)	0 (−0.08 to 0.08)	0.931	7,066,664	678.1 (474.4–924.1)	11,553,114	611.6 (426.6–836.1)	−0.14 (−0.23 to −0.05)	**0.003**
Western Sub-Saharan Africa	3,808,095	58971.4 (46872.9–74301.8)	7,605,285	59534.8 (47715.7–74207.8)	0.06 (−0.03 to 0.16)	0.185	3,808,095	2888.4 (2052.2–3923.7)	7,605,285	2686.5 (1898–3,677)	−0.19 (−0.42 to 0.04)	0.104

YLD, years lived with disability; ASPR, age-standardized prevalence rate; ASYR, age-standardized YLD rate; AAPC, average annual percentage changes. *P*-values less than 0.05 are considered statistically significant and are highlighted in bold.

**Table 2:** Global and sociodemographic index (SDI) quintile data on numbers for overall vision loss and age-standardized rates for specific causes of vision loss in 2019, with percentage changes from 1990 (age > =65 years).

	Prevalence number		Due to glaucoma		Due to cataract		Due to age-related macular degeneration		Due to refraction disorders		Due to near vision loss		Due to other causes	
Location	Number (UI)	Percentage change from 1990	Rate (UI)	Percentage change from 1990	Rate (UI)	Percentage change from 1990	Rate (UI)	Percentage change from 1990	Rate (UI)	Percentage change from 1990	Rate (UI)	Percentage change from 1990	Rate (UI)	Percentage change from 1990
Global	293674326.1 (235534978.8–368549406.2)	129.70%	874.8 (688–1087.1)	−15.76%	9729.4 (7970.1–11751.9)	6.95%	827.3 (645.3–1037.1)	−2.62%	6093.9 (4817–7,602)	−5.55%	29491.1 (19148.1–42899.6)	2.11%	2844.9 (2259–3566.2)	−5.43%
High SDI	25520118.3 (20798851.9–31388714.4)	93.60%	488 (381.9–612.6)	−9.19%	3003.2 (2313.9–3843.4)	−1.01%	446.4 (344.9–562.7)	−14.66%	2,743 (2106.6–3525.9)	−3.52%	6710.6 (4316.8–9,969)	3.59%	1096.4 (799.5–1478.5)	−5.3
High-middle SDI	77614557.5 (60904484.0–99308023.0)	108.52%	769.7 (605.4–959.4)	−18.74%	7624.4 (6062–9445.5)	14.81%	925.7 (718.7–1166.1)	4.22%	5620.8 (4394.7–7088.4)	−6.84%	31,571 (20604.6–45735.2)	−2.63%	2,986 (2355.9–3760.1)	−19.81%
Middel SDI	102118249.3 (81303185.5–129165833.6)	155.40%	1066.4 (840.6–1328.2)	−24.31%	12972.3 (10744–15456.2)	−5.94%	958.8 (747.2–1204.7)	−3.49%	6785.9 (5369.3–8,450)	−11.32%	37250.9 (23976.5–54705.5)	−7.93%	3796.9 (3066.3–4670.9)	−10.60%
Low-middle SDI	65087888.2 (52871879.6–80566062.5)	142.59%	1119.4 (881.7–1393.2)	−27.24%	17774.8 (14825–21077.8)	−10.86%	895.1 (699.6–1117.1)	−18.00%	10302.4 (8234.4–12694.1)	−18.44%	44487.8 (28774–65057.8)	−6.68%	3784.4 (3005.8–4,734)	−3.41%
Low SDI	23202943.7 (18818980.4–28578866.8)	119.50%	1753.6 (1378.2–2181.8)	−18.09%	15921.4 (13308–18866.7)	−1.23%	1145.0 (902.2–1423.1)	−1.72%	8239.6 (6593.5–10133.2)	−6.28%	49526.2 (33624.7–69192.3)	−4.84%	3680.3 (2906.1–4623.6)	5.01%

SDI, sociodemographic index.

**Table 3:** Global and sociodemographic index (SDI) quintile data on numbers of years lived with disability (YLDs) for overall vision loss and age-standardized rate for specific causes of vision loss YLDs in 2019, with percentage changes from 1990 (age > =65 years).

	Prevalence number		Due to glaucoma		Due to cataract		Due to age-related macular degeneration		Due to refraction disorders		Due to near vision loss		Due to other causes	
	Number (UI)	Percentage change from 1990	Rate (UI)	Percentage change from 1990	Rate (UI)	Percentage change from 1990	Rate (UI)	Percentage change from 1990	Rate (UI)	Percentage change from 1990	Rate (UI)	Percentage change from 1990	Rate (UI)	Percentage change from 1990
Global	11187620.2 (7845107.2–15390016.3)	110.21%	88.7 (58.3–129.9)	−25.40%	648.0 (451.4–890.2)	−7.72%	60.5 (40.3–86.7)	−14.79%	284.2 (188.9–405.3)	−8.29%	284.7 (121.9–565.8)	2.30%	212.9 (146–300.1)	−8.70%
High SDI	983828.4 (683795.4–1353356.3)	83.84%	50.5 (33.4–73.7)	−15.13%	162.1 (107.8–229.2)	−6.79%	38.8 (25.4–56.6)	−22.24%	121.6 (78.7–178.2)	−5.00%	64.6 (27.5–129.6)	3.69%	70.3 (46.5–101.1)	−8.10%
High-middle SDI	2459534.9 (1686538.8–3452585.7)	100.66%	78.2 (51.4–114.7)	−29.68%	440.9 (304–611.8)	1.08%	66.6 (44.4–95.3)	−12.14%	254.4 (167.3–367.7)	−5.39%	306.3 (130.6–610)	−2.17%	207.4 (142.6–289.9)	−20.32%
Middel SDI	3959524.8 (2781266.2–5452397.5)	126.88%	106.1 (69.8–156.2)	−35.58%	882.2 (616–1210.8)	−22.55%	65.1 (43.2–93)	−13.89%	329.8 (219.9–468.1)	−14.78%	359.5 (152.6–713.1)	−7.82%	303.3 (207.2–429.3)	−17.58%
Low-middle SDI	2818977.1 (1988172.2–3866223.9)	109.26%	110.5 (72.7–161.7)	−34.42%	1314.8 (920.5–1795.1)	−23.64%	64.5 (43.4–91.7)	−24.83%	479.6 (320.3–678.7)	−24.46%	425.2 (180.8–847.1)	−6.55%	293.5 (201.2–413.8)	−9.83%
Low SDI	960346.3 (679488.9–1320247.3)	106.00%	201.0 (130.4–296.7)	−22.69%	1230.7 (859.9–1695.8)	−13.46%	84.7 (57.2–120.1)	−9.41%	400.9 (269.1–564.6)	−10.85%	476.3 (207.2–926.8)	−4.61%	324.2 (219.7–468.3)	−2.17%

SDI, sociodemographic index; YLDs, years lived with disability.

### 3.2 Distribution of prevalence and YLDs by age and sex

In terms of sex, the global prevalence and YLD rates were all lower among males than among females (Table 1). For males, the prevalence rates remained relatively stable, while the YLD rates exhibited a significant decline with an AAPC of −0.28. In contrast, among females, the prevalence rates showed a slight increase with an AAPC of 0.19, while the YLD rates did not show any significant changes. Supplementary Tables S1–S7 showed the distribution of prevalence and YLDs of overall vision loss and specific causes of vision loss by gender and age subgroup. The prevalence of cataract and near vision loss among different age subgroups exhibited a consistent distribution pattern similar to the overall vision loss, with distinct upward trends among females in the age groups of 65–69 years, 70–74 years, 75–79 years, 80–84 years, and 85–89 years. Additionally, there was a distinct increasing trend in near vision loss among females in the 90–94 years age group. Regarding YLDs by age subgroup, there was no significant increase in overall vision loss across all age groups among females. However, cataract demonstrated an increase specifically in the 75–79 years age group, while near vision loss exhibited an elevated trend in the 65–69 years, 75–79 years, 80–84 years, and 90–94 years age groups. In contrast, the YLDs associated with the remaining specific causes of vision loss either experienced a significant decrease or showed no significant increase when examined by gender and age subgroup.

### 3.3 Burden trends by region

Among 21 GBD regions by SDI in 2019, East Asia had the highest vision loss prevalence, while Southern Sub-Saharan Africa had the highest ASPR per 100,000 population (67931.4, 95% UI: 51008.7–87105.2; Table 1). The North Africa and Middle East region experienced the most rapid decline in prevalence rates (AAPC = -0.34; 95% CI: −0.41 to −0.28). Regarding the YLDs due to overall vision loss, East Asia ranked first. Southern Sub-Saharan Africa had the highest ASPR per 100,000 population (3143.5; 95% UI: 2236.8–4268.4; Table 1). Southeast Asia exhibited the most rapid decrease rate in prevalence rates (AAPC = −1; 95% CI: −1.12 to −0.88). However, despite a significant downward trend in prevalence and YLDs observed in most regions, the High-income Asia Pacific region showed a significant increase in prevalence (AAPC = 0.2; 95% CI: 0.12 to 0.28; *p* < 0.001), while YLDs did not change significantly (*p* = 0.482).

### 3.4 Burden trends of overall vision loss by SDI

**Figure 3:**
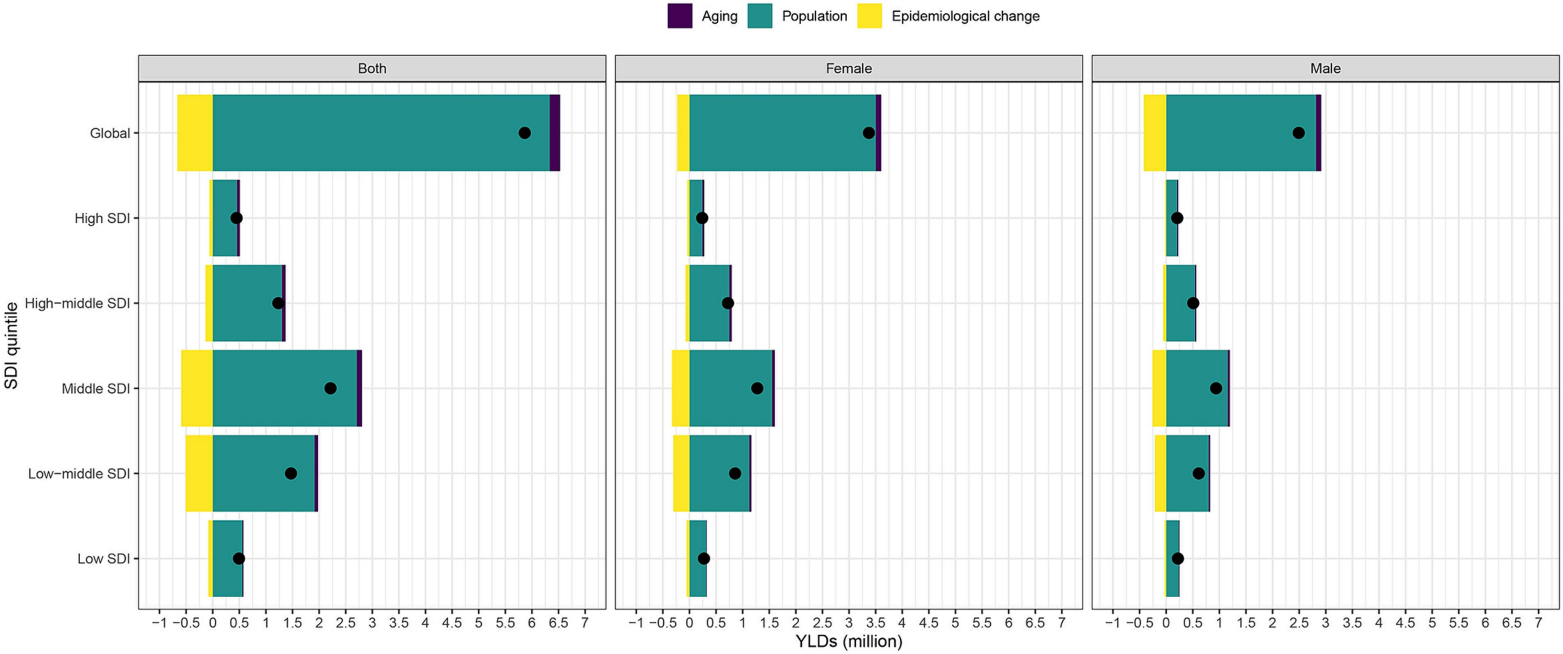
Changes in overall vision loss YLDs according to population-level determinants of population growth, aging and epidemiological change from 1990 to 2019 at the global level and by SDI quintile. The black dot represents the overall value of change contributed by all three components. For each component, the magnitude of a positive value indicates a corresponding increase in vision loss YLDs attributed to the component, and the magnitude of a negative value indicates a corresponding decrease in vision loss YLDs attributed to the related component. YLDs, years lived with disability, SDI, socio-demographic index.

Vision loss epidemiology is driven by population growth, aging, and epidemiological changes. Globally and within each SDI quintile, there was a significant increase in YLDs attributed to vision loss over the past 30 years. The most substantial increase in YLDs was observed in the Middle and Low-middle SDI quintiles (Figure 3). YLDs contributed most to aging in the Middle SDI quintile, while declining in the High SDI, High-middle SDI, Low-middle SDI, and Low SDI quintiles. A significant amount of changes in vision loss YLDs between SDI quintiles were associated with shifts in age and population, with population growth playing a larger role in Low-SDI and Low-middle-SDI countries.

**Figure 4:**
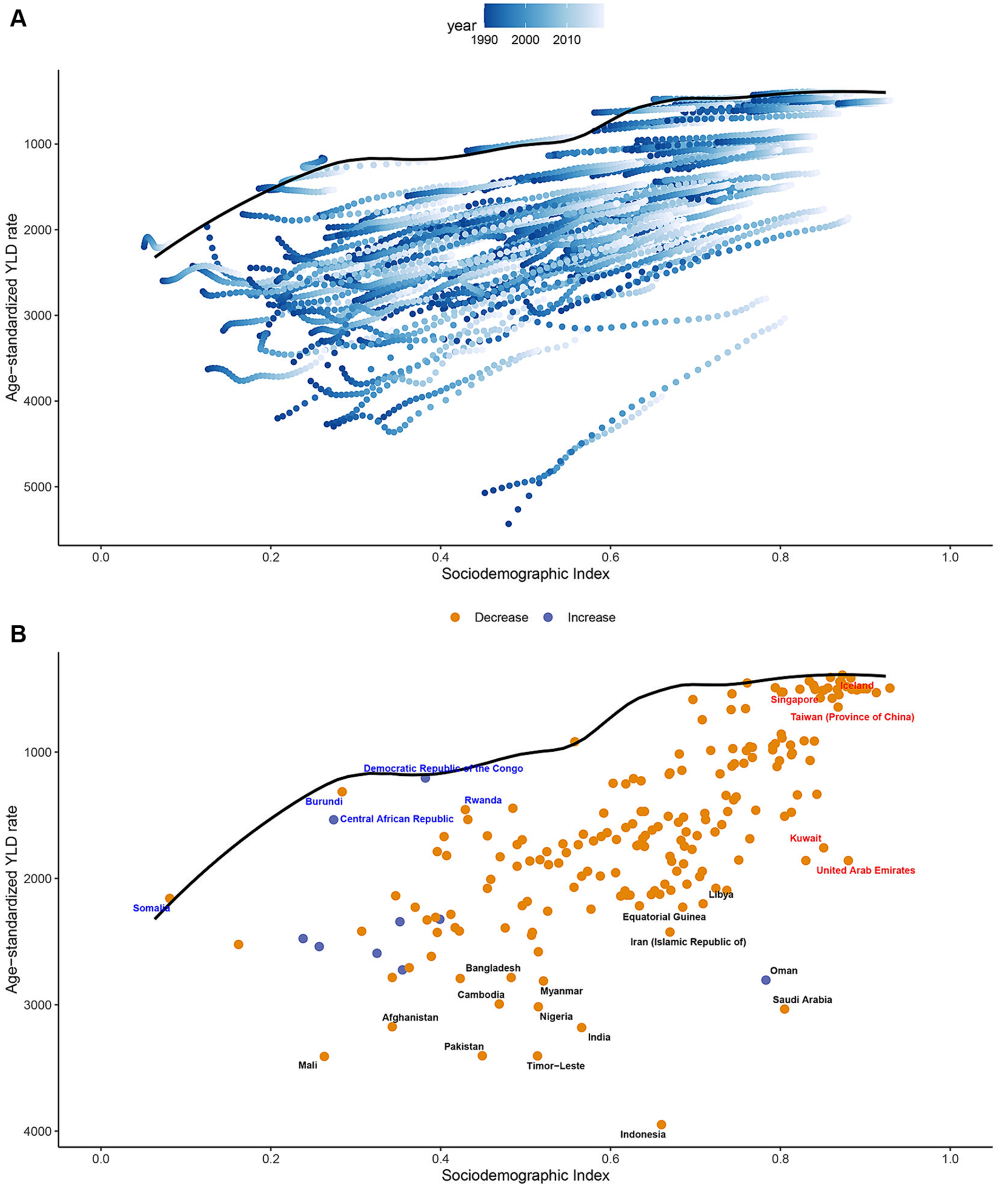
**(A)** Frontier analysis based on SDI and age-standardized overall vision loss YLDs rate from 1990 to 2019. The frontier is delineated in solid black color; countries and territories are represented as dots. **(B)** Frontier analysis based on SDI and age-standardized overall vision loss YLDs rate in 2019. The top 15 countries with the largest effective difference (largest overall vision loss YLDs gap from the frontier) are labeled in black; examples of frontier countries with low SDI (<0.5) and low effective difference are labeled in blue (e.g., Democratic Republic of the Congo, Rwanda, Burundi, Central African Republic and Somalia); and examples of countries and territories with high SDI (>0.85) and relatively high effective difference for their level of development are labeled in red (e.g., Iceland, Singapore, Taiwan, Kuwait and United Arab Emirates). Red dots indicate an increase in overall vision loss YLDs rate from 1990 to 2019; blue dots indicate a decrease in age-standardized overall vision loss YLDs rate between 1990 and 2019. YLDs, years lived with disability; SDI, socio-demographic index.

In order to gain a better understanding of the YLD rates of vision loss and a country’s development status, we built a frontier analysis based on age-standardized YLDs rates and SDI using data from 1990 to 2019 (Figure 4). The trends in vision loss YLDs and epidemiological changes varied across different GBD regions, with some regions experiencing decreases in age-standardized YLD rate and others showing increasing trends. Frontier lines indicate the areas with the lowest YLD rates (optimal performers) based on their SDI. A country’s effective distance from the frontier is defined as the gap between a country’s observed and potentially achievable YLDs; this gap can be reduced or eliminated based on the country or region’s sociodemographic resources. In 2019, the SDI and YLDs were used to calculate the effective difference between each country and region (Figure 4 and Supplementary Table S6). As SDI increased, the effective difference tended to be smaller and less variable.

### 3.5 Burden trends of specific causes of vision loss by SDI

Based on the predominant causes of vision loss related to population aging, we conducted a decomposition analysis and frontier analysis focusing on six key factors: glaucoma, cataracts, AMD, refractive disorders, near vision loss and other causes. Decomposition analysis of YLDs in specific causes of vision loss was shown in Supplementary Figure S1. Trends in the burden of cataract and near vision loss by SDI quintiles were consistent with trends in the burden of overall causes of vision loss, both showing the greatest contribution of YLDs to the aging of middle SDI quintiles with a gradual decrease on either side. Additionally, glaucoma and refraction disorders showed the greatest contribution of YLDs to the aging of high SDI quintiles, while AMD and other causes had the highest contribution in high-middle SDI quintiles, with a gradual decrease on either side. The frontier analysis demonstrates consistent characteristics of the frontier line, revealing that as SDI increases, the effective differences tend to decrease in size and become less variable (Supplementary Figure S2).

## 4 Discussion

This study comprehensively analyzed the prevalence rates and YLDs related to vision loss among individuals aged 65 years and older from 1990 to 2019. The research explored various factors, including the specific types of vision loss, different age groups, geographical regions, countries, and SDI quintiles. Our findings shed light on the complexities of vision loss in the geriatric population and underscore potential areas for targeted interventions across different demographics.

In 2019, there were 293.67 million cases of vision loss and 11.18 million YLDs worldwide. The prevalence increased and the YLD rate decreased, with AAPCs of 0.11 and − 0.12, respectively. Our findings are consistent with those of a previous report that conducted a systematic review and meta-analysis of population-based surveys on global vision impairment and blindness (22). Evaluating the increase in life expectancy during the study period, our analysis indicates a substantial increase in the number of cases of vision loss globally. This increase coincides with a notable rise in the global older adults population, indirectly reflecting the rising life expectancy and its implications on health system demands, including the need for enhanced vision care services. While our study did not directly analyze changes in life expectancy, the growing number of older adults individuals experiencing vision loss mirrors the implications of increased life expectancy on public health and vision care needs.

The rise in the prevalence of vision impairment can be attributed to a range of factors. A primary driver is the escalating demand for vision care services stemming from demographic shifts and changes in lifestyle (23). With the global population continuing to expand and age, the incidence of age-related vision conditions like cataracts, AMD and near vision loss is on the rise. Improved diagnostic capabilities and advancements in healthcare infrastructure have contributed to better detection and reporting of vision disorders in various regions. High-income and high-middle SDI regions are often home to populations with longer life expectancies (24, 25), leading to a greater number of individuals reaching an age where vision disorders are more prevalent. Additionally, lifestyle factors such as smoking (26–28) and exposure to sunlight (29–31) have been shown to increase the risk of cataracts and AMD, particularly in high-middle SDI regions like Eastern Europe and High-income Asia Pacific (32). In high SDI regions, shifts in lifestyles and habits such as prolonged screen time can contribute to an increased prevalence rate of near vision loss (33). Access to advanced healthcare systems in high SDI regions also plays a role in the higher prevalence rates. Individuals in these regions have better options for vision correction, such as eyeglasses, contact lenses, or refractive surgeries, which can result in more individuals seeking and receiving appropriate treatment for refractive error. Presbyopia, which occurs due to the natural age-related decline in the eyes’ focusing ability, manifests when the clarity of near vision becomes insufficient despite optimal correction for distance vision. To effectively address these challenges, we propose the integration of vision care into broader healthcare strategies aimed at the aging population, emphasizing the importance of preventive measures and accessible corrective solutions, such as eyeglasses and contact lenses.

Our analysis highlights a significant global increase in the prevalence of vision loss due to cataracts, with a 6.95% rise from 1990 to 2019. This trend underscores that, even with marked improvements in cataract surgery techniques and earlier interventions, the total cases of vision loss attributable to cataracts have climbed. This phenomenon can primarily be attributed to the surge in the global aging population, which has expanded more rapidly than the rate of medical advancements in treating cataracts. Additionally, our study reveals notable differences across various SDI quintiles, with high-middle SDI regions experiencing a 14.81% increase in cataract-related vision loss, highlighting substantial global disparities in the accessibility to and execution of modern cataract surgery techniques. In contrast, low SDI regions demonstrated a decrease of −1.23%, suggesting a critical role of broader factors such as healthcare access and diagnostic capabilities in managing cataract-related vision impairment. These findings illustrate the intricate relationship between demographic shifts, progress in medical technologies, and healthcare access in addressing the challenge of cataract-induced vision loss. Despite advancements in surgical methods facilitating earlier diagnoses and treatments, the overall rise in cataract cases emphasizes the urgent need for comprehensive public health strategies. Such strategies should extend beyond merely enhancing surgical access to include a wider spectrum of eye care services, ensuring they are accessible and affordable for the aging population worldwide. The increase in vision loss due to cataracts, despite the evolution of surgical interventions, necessitates policy initiatives aimed at augmenting global access to cataract surgery, particularly focusing on enhancing healthcare infrastructure in lower SDI regions. This approach is vital for mitigating the burden of vision loss on the aging demographic, ensuring equitable healthcare access, and fostering a healthier global community.

Notably, the GBD 2019 findings highlight that women exhibit higher prevalence rates and YLDs of vision loss compared to men. The increase in both prevalence and YLD rates of vision loss was greater among females than males. Possible factors contributing to this discrepancy include the postmenopausal decline in estrogen, which can thin the nerve fiber layer (34) and an elevated risk of cataracts (35), thereby increasing susceptibility to vision loss in women. The decline in estrogen levels may also have implications for ocular surface tissues and tear secretion (36), exerting additional effects on vision.

The study also highlights the significant burden of near vision impairment in the older population. In 2015, an estimated 666.7 million people aged 50 years or older experienced this condition (37). Additionally, a 2018 meta-analysis estimated that 826 million people had near vision impairment due to no or inadequate presbyopic correction (38). Based on the analysis from GBD 2019, it was estimated that there were approximately 250.2 million individuals aged 65 years and older who experienced near vision loss in 2019. It is noteworthy that both the prevalence of near vision loss and YLDs associated with it have been on the rise, particularly among women. Our analysis reveals a nuanced picture of vision loss due to refractive disorders, including near vision loss, which directly relates to the necessity for glasses. Globally, we observed a slight increase of 2.11% in the prevalence of vision loss due to near vision impairment from 1990 to 2019. This increase suggests that, despite advances in vision care and the availability of corrective lenses, the demand for and access to glasses has not fully mitigated the burden of vision loss attributable to refractive errors in the older adults population. The lack of a definition for near vision impairment in the International Classification of Diseases until 2019 limited the ability to provide a comprehensive analysis of temporal changes in the prevalence of vision impairment due to uncorrected presbyopia. Nonetheless, this underscores the importance of conducting surveys that incorporate a near vision component to gain better insights into future trends in this area.

Several limitations should be acknowledged in our study. Firstly, the reliance on the GBD 2019 dataset, while comprehensive, imposes constraints on our study’s granularity regarding specific causes of vision loss not classified within the main categories. Data availability varied across different world regions, leading to significant data gaps as previously described. Secondly, many studies were not conducted at a national level, and regional assessments were predominant for several countries. This may affect the uniformity and standardization of diagnostic tools and criteria used across different studies, given the diversity of healthcare systems and practices worldwide. Policy-making regarding vision impairment typically occurs at a national level, making national-level data more relevant. Furthermore, our study’s definitions and categorization of vision loss were aligned with the GBD 2019 criteria, which categorize vision impairment based on visual acuity in the better-seeing eye. This approach allowed us to cover a broad spectrum of vision impairment, from mild vision loss to complete blindness. However, it also meant that other significant causes of vision loss, such as diabetic retinopathy, stroke, and retinal detachment, were not included as primary focus areas due to their classification under “other causes” in the GBD framework. The issue of under-corrected presbyopia has often been overlooked, even in major ophthalmology studies, resulting in less precise estimates. Additionally, variations in measurement methods, such as objective versus functional presbyopia, test distance, and font size, further contribute to limitations in studies involving uncorrected presbyopia. In light of these concerns, we emphasize the need for future research to explore these other significant causes of vision loss more deeply, utilizing datasets that may offer more detailed categorization and diagnostic clarity. We are committed to enhancing the understanding of our study’s methodological rigor and the reliability of its findings, contributing valuable insights into the global burden of vision loss and its determinants.

Overall, this study provides valuable insights into the prevalence and impact of vision loss in the aging population, highlighting the need for comprehensive vision care services and interventions targeting different age groups and genders. Moreover, our discussion identifies future research directions focusing on lifestyle changes, early screening for age-related vision conditions, and the development of interventions tailored to mitigate identified risk factors.

## 5 Conclusion

The past three decades have witnessed remarkable advancements in mitigating the impact of blindness and vision loss among individuals aged 65 years and above worldwide. This encouraging trend is a testament to the progress made in eye care and public health initiatives. However, it is crucial to acknowledge that significant variations persist in the burden of these conditions, influenced by factors such as the type of impairment, a country’s SDI, and specific age groups.

The presence of age-specific variations in the burden of vision loss underscores the necessity for targeted interventions. Older adults often exhibit distinct risk profiles and have specific eye health needs compared to younger age groups. Tailoring screening programs, treatment protocols, and rehabilitation services to address the unique challenges faced by older adults can further reduce the burden of vision loss and enhance their overall quality of life.

In conclusion, while significant progress has been made in reducing the burden of blindness and vision loss among older adults, there is still work to be done. By implementing strategies that focus on improving screening coverage, ensuring quality control, addressing specific impairments like uncorrected presbyopia, and targeting interventions based on country SDIs and age groups, we can continue to make strides in reducing the burden of vision loss among older adults globally.

## Data availability statement

Data are available on the Global Health Data Exchange GBD 2019 website (https://ghdx.healthdata.org/gbd-2019). Both the statistical code and detailed region-or country-specific decomposition results of vision loss are available upon request from YW at wangyb35@csu.edu.cn.

## Author contributions

JY: Writing – review & editing, Investigation, Visualization. BJ: Data curation, Methodology, Writing – review & editing. TZ: Data curation, Methodology, Writing – review & editing. XG: Data curation, Methodology, Writing – review & editing. YT: Conceptualization, Data curation, Methodology, Visualization, Writing – review & editing. YW: Conceptualization, Formal analysis, Investigation, Project administration, Supervision, Writing – original draft.
