# Supplementary material for: Trends in the global burden of vision loss among the older adults from 1990 to 2019
Source: Front Public Health. 2024 Apr 4;12:1324141. doi: 10.3389/fpubh.2024.1324141 (PMC11025641; doi:10.3389/fpubh.2024.1324141)
Supplement: Supplementary file 1 [file Data_Sheet_1.DOCX]

**Supplementary Table 1.** **Prevalence and Years Lived with Disability (YLDs) of Overall Vision Loss and their average annual percentage changes (AAPCs) from 1990 to 2019 at the Global Level (Age>=65 Years)**

|  | Prevalence | | | |  |  |
| --- | --- | --- | --- | --- | --- | --- |
|  | case (n), 1990 | Prevalence (per 100,000 population), 1990 | case (n), 2019 | Prevalence (per 100,000 population), 2019 | AAPC, 1990-2019 | p value |
| Vision loss |  |  |  |  |  |  |
| Male |  |  |  |  |  |  |
| 65-69 years | 19035829.1 (14712056.1-24284059) | 33245.9 (25694.4-42411.8) | 41856707.9 (32357276.8-53438962.1) | 33860.1 (26175.5-43229.6) | 0.07 (0.02 to 0.11) | **0.002** |
| 70-74 years | 14596139 (11707893.9-18206121.3) | 38900.6 (31203.1-48521.7) | 33517133.5 (26846632.2-41931610.1) | 38042.8 (30471.6-47593.5) | -0.06 (-0.1 to -0.01) | **0.015** |
| 75-79 years | 10489467.8 (8533469.5-13248318.9) | 41860.6 (34054.8-52870.5) | 23926771.4 (19471064.9-30319554.1) | 41826.4 (34037.3-53001.6) | 0.04 (-0.03 to 0.1) | 0.263 |
| 80-84 years | 5953200.2 (4899089.5-7410102.2) | 45162.7 (37165.9-56215.2) | 15733672.6 (12889455.7-19901830.9) | 44650.1 (36578.5-56478.7) | 0 (-0.09 to 0.08) | 0.937 |
| 85-89 years | 2361896.8 (1973002.7-2854603) | 47493.8 (39673.8-57401.4) | 7487764 (6195223-9139948.2) | 45984.3 (38046.5-56130.8) | -0.1 (-0.17 to -0.03) | **0.005** |
| 90-94 years | 601725.8 (507669.2-712219.1) | 48423.5 (40854.3-57315.3) | 2475087.7 (2071978.9-2969372) | 46630.4 (39035.9-55942.7) | -0.12 (-0.19 to -0.05) | **0.001** |
| 95+ years | 126617.7 (105253.8-150023.5) | 49671.4 (41290.5-58853.4) | 604558 (495345.6-727217.6) | 47366.5 (38809.8-56976.7) | -0.14 (-0.24 to -0.05) | **0.004** |
| Female |  |  |  |  |  |  |
| 65-69 years | 23065284 (17848295.2-29330667.7) | 34825.6 (26948.6-44285.5) | 50410313.5 (39086724-64108603.9) | 37350.1 (28960.2-47499.5) | 0.25 (0.2 to 0.3) | **0** |
| 70-74 years | 19042474.7 (15295463.4-23556373) | 40523 (32549.2-50128.7) | 41170485.1 (33024021.9-51505155.9) | 41593.3 (33363.2-52034.1) | 0.09 (0.04 to 0.14) | **0** |
| 75-79 years | 15734697.2 (12821980.6-19831394.6) | 43404.7 (35369.9-54705.5) | 31822170 (25993577.9-40084508.5) | 45558.7 (37214.1-57387.6) | 0.18 (0.09 to 0.28) | **0** |
| 80-84 years | 10120370 (8350250.3-12494721.2) | 45922.4 (37890.3-56696.3) | 23971052.6 (19701276-29841238.8) | 48736.9 (40055.8-60672) | 0.23 (0.17 to 0.3) | **0** |
| 85-89 years | 4813748.7 (4031684.3-5775317.3) | 47681.3 (39934.8-57205.9) | 13363832.2 (11097254.9-16261235.2) | 49136.1 (40802.3-59789.2) | 0.12 (0.05 to 0.19) | **0.001** |
| 90-94 years | 1522573.2 (1293708.9-1779311.4) | 48131.3 (40896.5-56247.2) | 5660554.8 (4756133.2-6730946) | 49010.4 (41179.7-58278.1) | 0.06 (0 to 0.12) | 0.063 |
| 95+ years | 385941.1 (322839.5-450848.1) | 49828.7 (41681.7-58208.8) | 1674222.8 (1376902.1-1994623.7) | 47877.8 (39375.3-57040.3) | -0.13 (-0.16 to -0.1) | **0** |
|  |  |  |  |  |  |  |
|  | YLDs | | | |  |  |
|  | case (n), 1990 | YLDs (per 100,000 population), 1990 | case (n), 2019 | YLDs (per 100,000 population), 2019 | AAPC, 1990-2019 | p value |
| Vision loss |  |  |  |  |  |  |
| Male |  |  |  |  |  |  |
| 65-69 years | 672324.6 (467315.4-941437.8) | 1174.2 (816.2-1644.2) | 1316066 (900582.4-1868697.8) | 1064.6 (728.5-1511.7) | -0.3 (-0.41 to -0.2) | **0** |
| 70-74 years | 586887.6 (418581.2-804825.6) | 1564.1 (1115.6-2145) | 1200044.8 (837124.6-1657777.3) | 1362.1 (950.2-1881.6) | -0.45 (-0.59 to -0.3) | **0** |
| 75-79 years | 460682.5 (329800.5-629782.8) | 1838.5 (1316.1-2513.3) | 954493 (675818-1313139.8) | 1668.5 (1181.4-2295.5) | -0.3 (-0.37 to -0.24) | **0** |
| 80-84 years | 290723.4 (208086.5-391532.7) | 2205.5 (1578.6-2970.3) | 679580.2 (480685.3-918141.3) | 1928.6 (1364.1-2605.6) | -0.37 (-0.55 to -0.19) | **0** |
| 85-89 years | 129130.8 (93045.8-171401.7) | 2596.6 (1871-3446.6) | 358245.3 (255331.1-476427.3) | 2200.1 (1568.1-2925.9) | -0.51 (-0.62 to -0.4) | **0** |
| 90-94 years | 37067.5 (26503.4-48603.6) | 2983 (2132.8-3911.3) | 132630.2 (94178.7-175256) | 2498.7 (1774.3-3301.8) | -0.6 (-0.7 to -0.5) | **0** |
| 95+ years | 8582 (6088.9-11498.8) | 3366.7 (2388.6-4510.9) | 35976.6 (25431.5-48399.4) | 2818.7 (1992.5-3792) | -0.61 (-0.65 to -0.57) | **0** |
| Female |  |  |  |  |  |  |
| 65-69 years | 819771.8 (570867.6-1150963.7) | 1237.7 (861.9-1737.8) | 1609973.6 (1106414.2-2278223.3) | 1192.9 (819.8-1688) | -0.11 (-0.22 to 0) | **0.043** |
| 70-74 years | 776963.6 (554970.9-1065508.5) | 1653.4 (1181-2267.4) | 1519470 (1065799.4-2092729.8) | 1535.1 (1076.7-2114.2) | -0.23 (-0.36 to -0.1) | **0.001** |
| 75-79 years | 687635.7 (493865-941778.7) | 1896.9 (1362.3-2597.9) | 1305340.7 (928469.7-1791925.3) | 1868.8 (1329.3-2565.4) | -0.03 (-0.11 to 0.05) | 0.489 |
| 80-84 years | 484348.9 (344719.7-649727.2) | 2197.8 (1564.2-2948.2) | 1049143.1 (743256.9-1410238.1) | 2133.1 (1511.2-2867.2) | 0.01 (-0.21 to 0.23) | 0.952 |
| 85-89 years | 252984.6 (182132-336098.2) | 2505.9 (1804.1-3329.1) | 634828.3 (453006.9-844880.6) | 2334.1 (1665.6-3106.5) | -0.18 (-0.3 to -0.06) | **0.004** |
| 90-94 years | 89577.3 (63778.1-117956.1) | 2831.7 (2016.1-3728.8) | 293984.5 (209187.3-389988.4) | 2545.4 (1811.2-3376.6) | -0.34 (-0.44 to -0.23) | **0** |
| 95+ years | 25563.2 (18032.8-34186) | 3300.5 (2328.2-4413.7) | 97843.8 (69222.7-132258.5) | 2798 (1979.6-3782.2) | -0.55 (-0.61 to -0.5) | **0** |

YLDs, years lived with disability; AAPC, average annual percentage changes. p-values less than 0.05 are considered statistically significant and are highlighted in bold.
