# Supplementary material for: Trends in the global burden of vision loss among the older adults from 1990 to 2019
Source: Front Public Health. 2024 Apr 4;12:1324141. doi: 10.3389/fpubh.2024.1324141 (PMC11025641; doi:10.3389/fpubh.2024.1324141)
Supplement: Supplementary file 2 [file Data_Sheet_2.DOCX]

**Supplementary Table 2.** **Prevalence and Years Lived with Disability (YLDs) of Glaucoma and their average annual percentage changes (AAPCs) from 1990 to 2019 at the Global Level (Age>=65 Years)**

|  | Prevalence | | | |  |  |
| --- | --- | --- | --- | --- | --- | --- |
|  | case (n), 1990 | Prevalence (per 100,000 population), 1990 | case (n), 2019 | Prevalence (per 100,000 population), 2019 | AAPC, 1990-2019 | p value |
| Glaucoma |  |  |  |  |  |  |
| Male |  |  |  |  |  |  |
| 65-69 years | 343234.2 (267865.9-428700.9) | 599.5 (467.8-748.7) | 609591.3 (473507.8-765099.9) | 493.1 (383-618.9) | -0.64 (-0.72 to -0.56) | **0** |
| 70-74 years | 362148.3 (288487.9-448247.1) | 965.2 (768.9-1194.6) | 664295.5 (527191-826990.3) | 754 (598.4-938.7) | -0.76 (-0.92 to -0.6) | **0** |
| 75-79 years | 345691.8 (277537.6-425494.4) | 1379.6 (1107.6-1698) | 642307.6 (514481.9-793248.4) | 1122.8 (899.4-1386.7) | -0.68 (-0.75 to -0.6) | **0** |
| 80-84 years | 255724 (199382.1-316360.8) | 1940 (1512.6-2400) | 546018.8 (423825.1-672690.7) | 1549.5 (1202.8-1909) | -0.72 (-0.83 to -0.61) | **0** |
| 85-89 years | 133605.6 (105807-165404.3) | 2686.6 (2127.6-3326) | 345957 (277600.2-425231.6) | 2124.6 (1704.8-2611.5) | -0.78 (-0.85 to -0.7) | **0** |
| 90-94 years | 42361.4 (33652.7-52885.8) | 3409 (2708.2-4255.9) | 142711.3 (114156.9-177135.2) | 2688.7 (2150.7-3337.2) | -0.81 (-0.84 to -0.77) | **0** |
| 95+ years | 10956.2 (8496.7-14179.1) | 4298 (3333.2-5562.4) | 44972.8 (35127.7-57463.3) | 3523.6 (2752.2-4502.2) | -0.67 (-0.71 to -0.64) | **0** |
| Female |  |  |  |  |  |  |
| 65-69 years | 285059.8 (219972.7-356306.8) | 430.4 (332.1-538) | 532876.5 (411139.4-670971.5) | 394.8 (304.6-497.1) | -0.28 (-0.36 to -0.2) | **0** |
| 70-74 years | 321137 (255066.5-404083.9) | 683.4 (542.8-859.9) | 589664.4 (467159.7-738035.4) | 595.7 (472-745.6) | -0.41 (-0.55 to -0.27) | **0** |
| 75-79 years | 354927.2 (281842.5-438043.5) | 979.1 (777.5-1208.4) | 609398.3 (484679.5-755126.8) | 872.5 (693.9-1081.1) | -0.35 (-0.41 to -0.29) | **0** |
| 80-84 years | 304306.2 (234662.8-381072.8) | 1380.8 (1064.8-1729.2) | 583195.7 (448977-722575.4) | 1185.7 (912.8-1469.1) | -0.48 (-0.58 to -0.38) | **0** |
| 85-89 years | 192879.1 (151396.3-240707.6) | 1910.5 (1499.6-2384.3) | 429907.6 (338298.5-531742.1) | 1580.7 (1243.9-1955.1) | -0.63 (-0.69 to -0.58) | **0** |
| 90-94 years | 76839.3 (60336.9-96464.1) | 2429 (1907.4-3049.4) | 227053.8 (179257.8-283939.3) | 1965.9 (1552.1-2458.4) | -0.72 (-0.76 to -0.67) | **0** |
| 95+ years | 24606.4 (18994.3-31914.5) | 3176.9 (2452.3-4120.5) | 92785 (72005.8-119688) | 2653.4 (2059.2-3422.7) | -0.61 (-0.65 to -0.58) | **0** |
|  |  |  |  |  |  |  |
|  | YLDs | | | |  |  |
|  | case (n), 1990 | YLDs (per 100,000 population), 1990 | case (n), 2019 | YLDs (per 100,000 population), 2019 | AAPC, 1990-2019 | p value |
| Glaucoma |  |  |  |  |  |  |
| Male |  |  |  |  |  |  |
| 65-69 years | 39860.3 (25665.8-59441.7) | 69.6 (44.8-103.8) | 59930 (38707-89488.9) | 48.5 (31.3-72.4) | -1.21 (-1.32 to -1.11) | **0** |
| 70-74 years | 42474.6 (27868.9-62291.6) | 113.2 (74.3-166) | 66931.5 (43612.8-98995.1) | 76 (49.5-112.4) | -1.29 (-1.43 to -1.15) | **0** |
| 75-79 years | 40881.7 (26632.8-61051.9) | 163.1 (106.3-243.6) | 66805.9 (44285.4-98511.4) | 116.8 (77.4-172.2) | -1.12 (-1.24 to -1.01) | **0** |
| 80-84 years | 30380.3 (19997.2-44855.5) | 230.5 (151.7-340.3) | 58395.6 (38713.7-85043.6) | 165.7 (109.9-241.3) | -1.09 (-1.19 to -0.98) | **0** |
| 85-89 years | 16040.9 (10591.6-23424.3) | 322.6 (213-471) | 38230.2 (25373.9-55176.8) | 234.8 (155.8-338.9) | -1.06 (-1.13 to -0.99) | **0** |
| 90-94 years | 5140.7 (3378.5-7318.6) | 413.7 (271.9-589) | 16198.5 (10807.1-22871.1) | 305.2 (203.6-430.9) | -1.04 (-1.09 to -0.98) | **0** |
| 95+ years | 1336.3 (862.9-1970.1) | 524.2 (338.5-772.9) | 5204.9 (3404.5-7659.2) | 407.8 (266.7-600.1) | -0.85 (-0.88 to -0.82) | **0** |
| Female |  |  |  |  |  |  |
| 65-69 years | 30759.8 (19721.9-46049.5) | 46.4 (29.8-69.5) | 48707 (31498.7-71790.9) | 36.1 (23.3-53.2) | -0.84 (-0.91 to -0.76) | **0** |
| 70-74 years | 35202.9 (22972.7-51765) | 74.9 (48.9-110.2) | 55803 (36598.3-81725.3) | 56.4 (37-82.6) | -0.91 (-1.04 to -0.77) | **0** |
| 75-79 years | 39397.8 (25583.8-59560.5) | 108.7 (70.6-164.3) | 59674.2 (39603.8-88276.9) | 85.4 (56.7-126.4) | -0.81 (-0.9 to -0.72) | **0** |
| 80-84 years | 34001.1 (22210.9-50424) | 154.3 (100.8-228.8) | 58369.1 (38371.5-85480.2) | 118.7 (78-173.8) | -0.85 (-0.96 to -0.73) | **0** |
| 85-89 years | 21749.4 (14161-31693.8) | 215.4 (140.3-313.9) | 44211.1 (29002.5-63959.5) | 162.6 (106.6-235.2) | -0.94 (-1.01 to -0.87) | **0** |
| 90-94 years | 8692.6 (5723.5-12523) | 274.8 (180.9-395.9) | 23714.8 (15763.6-34012.5) | 205.3 (136.5-294.5) | -0.98 (-1.04 to -0.91) | **0** |
| 95+ years | 2808.9 (1813.3-4172.9) | 362.7 (234.1-538.8) | 9935.3 (6453.5-14808.8) | 284.1 (184.6-423.5) | -0.83 (-0.89 to -0.78) | **0** |

YLDs, years lived with disability; AAPC, average annual percentage changes. p-values less than 0.05 are considered statistically significant and are highlighted in bold.
