# Supplementary material for: Trends in the global burden of vision loss among the older adults from 1990 to 2019
Source: Front Public Health. 2024 Apr 4;12:1324141. doi: 10.3389/fpubh.2024.1324141 (PMC11025641; doi:10.3389/fpubh.2024.1324141)
Supplement: Supplementary file 3 [file Data_Sheet_3.DOCX]

**Supplementary Table 3.** **Prevalence and Years Lived with Disability (YLDs) of Cataract and their average annual percentage changes (AAPCs) from 1990 to 2019 at the Global Level (Age>=65 Years)**

|  | Prevalence | | | |  |  |
| --- | --- | --- | --- | --- | --- | --- |
|  | case (n), 1990 | Prevalence (per 100,000 population), 1990 | case (n), 2019 | Prevalence (per 100,000 population), 2019 | AAPC, 1990-2019 | p value |
| Cataract |  |  |  |  |  |  |
| Male |  |  |  |  |  |  |
| 65-69 years | 2669160.2 (2145984.4-3260517) | 4661.7 (3747.9-5694.5) | 6036831 (4744646.3-7511918.5) | 4883.5 (3838.2-6076.8) | 0.22 (0.07 to 0.37) | **0.004** |
| 70-74 years | 2766707.7 (2255985.4-3383613.6) | 7373.6 (6012.5-9017.8) | 6582555.1 (5305156.3-8096056.8) | 7471.4 (6021.5-9189.2) | 0.1 (-0.26 to 0.46) | 0.594 |
| 75-79 years | 2457548.4 (2059124.2-2911460.2) | 9807.4 (8217.4-11618.9) | 6004106.3 (4994608.5-7137669.5) | 10495.8 (8731.1-12477.4) | 0.28 (0.17 to 0.38) | **0** |
| 80-84 years | 1727287.4 (1432468.5-2078723.6) | 13103.7 (10867.1-15769.8) | 4658975.1 (3824174.4-5656898.4) | 13221.5 (10852.5-16053.5) | 0.2 (-0.1 to 0.49) | 0.198 |
| 85-89 years | 831121.7 (698484-971097.3) | 16712.5 (14045.4-19527.2) | 2619505.3 (2183509.1-3091578) | 16087.1 (13409.5-18986.2) | -0.05 (-0.19 to 0.1) | 0.511 |
| 90-94 years | 235984.2 (197750.6-278220.6) | 18990.7 (15913.8-22389.6) | 932379.9 (775609.9-1106043.6) | 17565.9 (14612.4-20837.8) | -0.27 (-0.32 to -0.22) | **0** |
| 95+ years | 52675.3 (42699.6-63701.9) | 20664.2 (16750.8-24989.9) | 240645.9 (192101.6-294006.6) | 18854.4 (15051-23035.1) | -0.3 (-0.35 to -0.25) | **0** |
| Female |  |  |  |  |  |  |
| 65-69 years | 3560700.1 (2890274.8-4318582.4) | 5376.2 (4363.9-6520.5) | 8202133 (6506744.7-10067111.8) | 6077.1 (4821-7458.9) | 0.49 (0.35 to 0.64) | **0** |
| 70-74 years | 3915893.8 (3231019.7-4734201.1) | 8333.1 (6875.7-10074.5) | 9005883.6 (7334208.4-10997205.2) | 9098.4 (7409.5-11110.1) | 0.35 (0.1 to 0.6) | **0.006** |
| 75-79 years | 3807910.9 (3202021.7-4495672) | 10504.2 (8832.9-12401.5) | 8627474.1 (7249697-10193524.5) | 12351.7 (10379.1-14593.7) | 0.59 (0.46 to 0.72) | **0** |
| 80-84 years | 2961750 (2447181-3555700.7) | 13439.3 (11104.4-16134.4) | 7455989.8 (6176655.4-9010508) | 15159.2 (12558.1-18319.8) | 0.6 (0.24 to 0.97) | **0.001** |
| 85-89 years | 1681073.2 (1412519.2-1974146.8) | 16651.4 (13991.3-19554.4) | 4833546.8 (4040080.1-5685562.2) | 17772 (14854.5-20904.6) | 0.3 (0.16 to 0.43) | **0** |
| 90-94 years | 611793.1 (511994.9-721927.8) | 19339.9 (16185.1-22821.4) | 2231415.6 (1866822.1-2639381.7) | 19320.1 (16163.4-22852.4) | 0.03 (-0.06 to 0.13) | 0.493 |
| 95+ years | 171155.9 (138829.3-205726) | 22097.9 (17924.2-26561.2) | 738383 (595731-895521.1) | 21115.6 (17036.1-25609.2) | -0.12 (-0.18 to -0.06) | **0** |
|  |  |  |  |  |  |  |
|  | YLDs | | | |  |  |
|  | case (n), 1990 | YLDs (per 100,000 population), 1990 | case (n), 2019 | YLDs (per 100,000 population), 2019 | AAPC, 1990-2019 | p value |
| Cataract |  |  |  |  |  |  |
| Male |  |  |  |  |  |  |
| 65-69 years | 550437.4 (373426.5-765916.7) | 373.9 (256.3-530.9) | 402279.8 (272913.2-563867) | 325.4 (220.8-456.1) | -0.43 (-0.64 to -0.22) | **0** |
| 70-74 years | 618001.9 (435534.3-856974) | 585.8 (409.3-825) | 438298.6 (307275.6-608331.5) | 497.5 (348.8-690.5) | -0.52 (-0.8 to -0.23) | **0** |
| 75-79 years | 594086.7 (416513.9-820926.1) | 757.8 (530.6-1055.9) | 397348.5 (277994.7-552741.9) | 694.6 (486-966.2) | -0.27 (-0.37 to -0.16) | **0** |
| 80-84 years | 501588.5 (353154.7-682548.7) | 993.2 (692.4-1357.8) | 303304.2 (211462.7-415490.1) | 860.7 (600.1-1179.1) | -0.32 (-0.63 to -0.01) | **0.04** |
| 85-89 years | 316304.4 (223889.9-420994) | 1248 (882.5-1687.8) | 169091.9 (119717.3-226393.5) | 1038.4 (735.2-1390.3) | -0.53 (-0.7 to -0.35) | **0** |
| 90-94 years | 138670.1 (97365.1-182351.7) | 1397.4 (972.4-1855.6) | 59612.3 (41590-79449.8) | 1123.1 (783.6-1496.8) | -0.77 (-0.94 to -0.6) | **0** |
| 95+ years | 43889.7 (29693.1-60471.4) | 1481.4 (996.4-2039.1) | 15029.5 (9910.8-20630.2) | 1177.5 (776.5-1616.4) | -0.78 (-0.84 to -0.72) | **0** |
| Female |  |  |  |  |  |  |
| 65-69 years | 285726.1 (198249.4-404909.7) | 431.4 (299.3-611.4) | 550437.4 (373426.5-765916.7) | 407.8 (276.7-567.5) | -0.13 (-0.35 to 0.09) | 0.238 |
| 70-74 years | 317452 (220285.2-436902.2) | 675.5 (468.8-929.7) | 618001.9 (435534.3-856974) | 624.3 (440-865.8) | -0.23 (-0.43 to -0.02) | **0.029** |
| 75-79 years | 298061.5 (208359-414979.5) | 822.2 (574.8-1144.7) | 594086.7 (416513.9-820926.1) | 850.5 (596.3-1175.3) | 0.16 (0.04 to 0.27) | **0.006** |
| 80-84 years | 223608.9 (156890-302550.6) | 1014.7 (711.9-1372.9) | 501588.5 (353154.7-682548.7) | 1019.8 (718-1387.7) | 0.24 (-0.18 to 0.67) | 0.263 |
| 85-89 years | 121748.2 (86816.2-162845.3) | 1205.9 (859.9-1613) | 316304.4 (223889.9-420994) | 1163 (823.2-1547.9) | -0.02 (-0.22 to 0.17) | 0.828 |
| 90-94 years | 42273.5 (29539.4-55858.8) | 1336.3 (933.8-1765.8) | 138670.1 (97365.1-182351.7) | 1200.6 (843-1578.8) | -0.27 (-0.56 to 0.03) | 0.078 |
| 95+ years | 11436.9 (7785.9-15620.7) | 1476.6 (1005.2-2016.8) | 43889.7 (29693.1-60471.4) | 1255.1 (849.1-1729.3) | -0.54 (-0.67 to -0.41) | **0** |

YLDs, years lived with disability; AAPC, average annual percentage changes. p-values less than 0.05 are considered statistically significant and are highlighted in bold.
