# Supplementary material for: Trends in the global burden of vision loss among the older adults from 1990 to 2019
Source: Front Public Health. 2024 Apr 4;12:1324141. doi: 10.3389/fpubh.2024.1324141 (PMC11025641; doi:10.3389/fpubh.2024.1324141)
Supplement: Supplementary file 4 [file Data_Sheet_4.DOCX]

**Supplementary Table 4.** **Prevalence and Years Lived with Disability (YLDs) of Age-related Macular Degeneration (AMD) and their average annual percentage changes (AAPCs) from 1990 to 2019 at the Global Level (Age>=65 Years)**

|  | Prevalence | | | |  |  |
| --- | --- | --- | --- | --- | --- | --- |
|  | case (n), 1990 | Prevalence (per 100,000 population), 1990 | case (n), 2019 | Prevalence (per 100,000 population), 2019 | AAPC, 1990-2019 | p value |
| AMD |  |  |  |  |  |  |
| Male |  |  |  |  |  |  |
| 65-69 years | 304887.9 (235460.3-380167.1) | 532.5 (411.2-664) | 669841.3 (515555.5-853645.7) | 541.9 (417.1-690.6) | 0.07 (0 to 0.14) | 0.056 |
| 70-74 years | 267265.9 (208088.4-334594.4) | 712.3 (554.6-891.7) | 622197.9 (478703.3-788010.7) | 706.2 (543.3-894.4) | -0.03 (-0.12 to 0.05) | 0.442 |
| 75-79 years | 213484.1 (168412.1-264768.6) | 852 (672.1-1056.6) | 492714.5 (388893.3-613356.2) | 861.3 (679.8-1072.2) | 0.01 (-0.11 to 0.13) | 0.904 |
| 80-84 years | 134244.9 (103954.2-167456.4) | 1018.4 (788.6-1270.4) | 351132.9 (272082.4-445084.3) | 996.5 (772.1-1263.1) | -0.09 (-0.25 to 0.07) | 0.276 |
| 85-89 years | 60086.3 (48221.1-73364.7) | 1208.2 (969.6-1475.2) | 185361.9 (147853.8-228932.4) | 1138.4 (908-1405.9) | -0.21 (-0.4 to -0.02) | **0.028** |
| 90-94 years | 16974.1 (13179.8-21425.4) | 1366 (1060.6-1724.2) | 64573.6 (50062.7-81165.6) | 1216.6 (943.2-1529.2) | -0.42 (-0.49 to -0.35) | **0** |
| 95+ years | 4053.1 (3061.4-5281.2) | 1590 (1201-2071.8) | 17848 (13447.3-23170.7) | 1398.4 (1053.6-1815.4) | -0.42 (-0.52 to -0.32) | **0** |
| Female |  |  |  |  |  |  |
| 65-69 years | 383941.6 (296403.9-477483.8) | 579.7 (447.5-720.9) | 828395.1 (639849.1-1043805.5) | 613.8 (474.1-773.4) | 0.19 (0.08 to 0.3) | **0** |
| 70-74 years | 365074.2 (287277.4-451711.2) | 776.9 (611.3-961.3) | 775460.7 (598521.9-982363.8) | 783.4 (604.7-992.5) | -0.02 (-0.17 to 0.13) | 0.821 |
| 75-79 years | 344764.3 (273673.9-423402.7) | 951 (754.9-1168) | 663168.3 (525317.2-817960.5) | 949.4 (752.1-1171) | -0.07 (-0.24 to 0.09) | 0.391 |
| 80-84 years | 265143.6 (208380.6-328354) | 1203.1 (945.6-1489.9) | 551125.2 (433455.8-689128.2) | 1120.5 (881.3-1401.1) | -0.29 (-0.39 to -0.18) | **0** |
| 85-89 years | 154397 (123958.9-188371.7) | 1529.3 (1227.8-1865.9) | 367779.1 (294271.2-446791.6) | 1352.2 (1082-1642.8) | -0.44 (-0.57 to -0.31) | **0** |
| 90-94 years | 59347.8 (46692.7-74522) | 1876.1 (1476-2355.8) | 182719 (143006.8-230258.3) | 1582 (1238.2-1993.6) | -0.62 (-0.78 to -0.45) | **0** |
| 95+ years | 18055.8 (13741.4-23548.4) | 2331.2 (1774.2-3040.3) | 69498.2 (53125.6-90396) | 1987.4 (1519.2-2585.1) | -0.55 (-0.63 to -0.47) | **0** |
|  |  |  |  |  |  |  |
|  | YLDs | | | |  |  |
|  | case (n), 1990 | YLDs (per 100,000 population), 1990 | case (n), 2019 | YLDs (per 100,000 population), 2019 | AAPC, 1990-2019 | p value |
| AMD |  |  |  |  |  |  |
| Male |  |  |  |  |  |  |
| 65-69 years | 23063.6 (15326.4-33418.2) | 40.3 (26.8-58.4) | 43684.5 (29052.6-62662.7) | 35.3 (23.5-50.7) | -0.43 (-0.54 to -0.31) | **0** |
| 70-74 years | 20189.4 (13584.6-28810) | 53.8 (36.2-76.8) | 41413.9 (27711.7-59222.3) | 47 (31.5-67.2) | -0.47 (-0.74 to -0.2) | **0.001** |
| 75-79 years | 16368.8 (10796.1-23789.4) | 65.3 (43.1-94.9) | 33560.9 (22195.3-48345.6) | 58.7 (38.8-84.5) | -0.39 (-0.51 to -0.27) | **0** |
| 80-84 years | 10589.1 (7119.8-14983.9) | 80.3 (54-113.7) | 24762.4 (16527.2-34963.2) | 70.3 (46.9-99.2) | -0.46 (-0.62 to -0.3) | **0** |
| 85-89 years | 4979.9 (3343-7038.3) | 100.1 (67.2-141.5) | 14078.9 (9485.6-19770.4) | 86.5 (58.3-121.4) | -0.5 (-0.57 to -0.44) | **0** |
| 90-94 years | 1519.5 (1006.7-2206.6) | 122.3 (81-177.6) | 5448.5 (3622.1-7886.5) | 102.7 (68.2-148.6) | -0.61 (-0.71 to -0.51) | **0** |
| 95+ years | 392.4 (242.4-594.8) | 153.9 (95.1-233.3) | 1648.2 (1018.5-2495.7) | 129.1 (79.8-195.5) | -0.58 (-0.68 to -0.47) | **0** |
| Female |  |  |  |  |  |  |
| 65-69 years | 30848.2 (20244.1-44679.8) | 46.6 (30.6-67.5) | 56738.2 (37786.8-81720.6) | 42 (28-60.5) | -0.36 (-0.49 to -0.23) | **0** |
| 70-74 years | 29653.9 (19998.6-42801.7) | 63.1 (42.6-91.1) | 55219.1 (37234.1-79095) | 55.8 (37.6-79.9) | -0.45 (-0.77 to -0.12) | **0.007** |
| 75-79 years | 29142.7 (19360.2-42799) | 80.4 (53.4-118.1) | 49234.5 (32808.5-71553.2) | 70.5 (47-102.4) | -0.5 (-0.61 to -0.39) | **0** |
| 80-84 years | 23623.7 (15857.6-34473.6) | 107.2 (72-156.4) | 43015.9 (28968-61013.9) | 87.5 (58.9-124.1) | -0.69 (-0.89 to -0.5) | **0** |
| 85-89 years | 14638.2 (9851.7-20774.9) | 145 (97.6-205.8) | 31203.7 (20922.3-43775.8) | 114.7 (76.9-161) | -0.86 (-1.1 to -0.62) | **0** |
| 90-94 years | 6011.7 (3963.1-8760.3) | 190 (125.3-276.9) | 16950.3 (11272.9-24517.8) | 146.8 (97.6-212.3) | -0.92 (-1.09 to -0.74) | **0** |
| 95+ years | 1948.5 (1210-2978.3) | 251.6 (156.2-384.5) | 7049.2 (4351.7-10689.1) | 201.6 (124.4-305.7) | -0.75 (-0.88 to -0.62) | **0** |

YLDs, years lived with disability; AMD, age-related macular degeneration; AAPC, average annual percentage changes. p-values less than 0.05 are considered statistically significant and are highlighted in bold.
