# Supplementary material for: Trends in the global burden of vision loss among the older adults from 1990 to 2019
Source: Front Public Health. 2024 Apr 4;12:1324141. doi: 10.3389/fpubh.2024.1324141 (PMC11025641; doi:10.3389/fpubh.2024.1324141)
Supplement: Supplementary file 5 [file Data_Sheet_5.DOCX]

**Supplementary Table 5.** **Prevalence and Years Lived with Disability (YLDs) of Refraction Disorders and their average annual percentage changes (AAPCs) from 1990 to 2019 at the Global Level (Age>=65 Years)**

|  | Prevalence | | | |  |  |
| --- | --- | --- | --- | --- | --- | --- |
|  | case (n), 1990 | Prevalence (per 100,000 population), 1990 | case (n), 2019 | Prevalence (per 100,000 population), 2019 | AAPC, 1990-2019 | p value |
| Refraction disorders |  |  |  |  |  |  |
| Male |  |  |  |  |  |  |
| 65-69 years | 3285304 (2612856-4038265.9) | 5737.7 (4563.3-7052.8) | 6647774.9 (5271223.6-8210173.8) | 5377.7 (4264.2-6641.6) | -0.23 (-0.28 to -0.17) | **0** |
| 70-74 years | 2463135.8 (1946121.2-3076775) | 6564.6 (5186.7-8200) | 5300344.1 (4160356.8-6698139.8) | 6016 (4722.1-7602.6) | -0.28 (-0.35 to -0.22) | **0** |
| 75-79 years | 1739421.5 (1418872.6-2141560.3) | 6941.6 (5662.3-8546.4) | 3748141.5 (3042837-4606598.2) | 6552.1 (5319.2-8052.8) | -0.23 (-0.4 to -0.05) | **0.012** |
| 80-84 years | 947264.4 (744746.8-1206357.2) | 7186.2 (5649.9-9151.8) | 2333409.1 (1819898.7-2976841.2) | 6621.9 (5164.6-8447.9) | -0.29 (-0.32 to -0.26) | **0** |
| 85-89 years | 337895.2 (268604-418684.7) | 6794.5 (5401.2-8419.1) | 1035250 (817995.3-1285123.8) | 6357.7 (5023.5-7892.3) | -0.22 (-0.34 to -0.1) | **0** |
| 90-94 years | 71078.4 (55676.4-91302.9) | 5720 (4480.5-7347.5) | 292662.4 (229691.7-374973.1) | 5513.7 (4327.4-7064.5) | -0.1 (-0.17 to -0.03) | **0.003** |
| 95+ years | 12157.3 (8751.3-16243.2) | 4769.2 (3433.1-6372.1) | 57759.6 (41754.7-77113.3) | 4525.4 (3271.4-6041.7) | -0.17 (-0.26 to -0.08) | **0** |
| Female |  |  |  |  |  |  |
| 65-69 years | 3918178.2 (3131830.6-4787467.1) | 5915.9 (4728.7-7228.5) | 7723219.3 (6108189-9525168.9) | 5722.3 (4525.7-7057.4) | -0.08 (-0.2 to 0.04) | 0.217 |
| 70-74 years | 3125713.6 (2474971-3875922.4) | 6651.6 (5266.8-8248.1) | 6173929.7 (4852213-7756385.7) | 6237.3 (4902-7836) | -0.22 (-0.3 to -0.14) | **0** |
| 75-79 years | 2540974 (2073678.2-3126195.9) | 7009.4 (5720.3-8623.7) | 4681678.1 (3804060.9-5765525.7) | 6702.6 (5446.1-8254.3) | -0.17 (-0.28 to -0.06) | **0.002** |
| 80-84 years | 1565762.5 (1228754.6-1986371.7) | 7104.8 (5575.6-9013.4) | 3357239 (2614020.4-4277065.4) | 6825.8 (5314.7-8695.9) | -0.11 (-0.2 to -0.02) | **0.021** |
| 85-89 years | 676552.6 (533641-847503.2) | 6701.4 (5285.8-8394.7) | 1744726 (1369594.9-2178194.3) | 6415 (5035.7-8008.8) | -0.15 (-0.23 to -0.07) | **0** |
| 90-94 years | 183524.1 (143031.1-236721.5) | 5801.5 (4521.5-7483.2) | 642913.4 (498360.2-823681) | 5566.5 (4314.9-7131.6) | -0.14 (-0.16 to -0.11) | **0** |
| 95+ years | 38804.3 (27902.9-52112.1) | 5010 (3602.5-6728.2) | 165163 (118937.8-223849.8) | 4723.2 (3401.3-6401.4) | -0.2 (-0.22 to -0.18) | **0** |
|  |  |  |  |  |  |  |
|  | YLDs | | | |  |  |
|  | case (n), 1990 | YLDs (per 100,000 population), 1990 | case (n), 2019 | YLDs (per 100,000 population), 2019 | AAPC, 1990-2019 | p value |
| Refraction disorders |  |  |  |  |  |  |
| Male |  |  |  |  |  |  |
| 65-69 years | 153509.6 (102252.1-218103.6) | 268.1 (178.6-380.9) | 297606.4 (195541.9-427769.8) | 240.7 (158.2-346) | -0.29 (-0.55 to -0.02) | **0.032** |
| 70-74 years | 119209.4 (79875.2-170636.7) | 317.7 (212.9-454.8) | 244680.9 (163307.9-350476.6) | 277.7 (185.4-397.8) | -0.41 (-0.54 to -0.27) | **0** |
| 75-79 years | 84697.4 (57653.4-118742.1) | 338 (230.1-473.9) | 176960.1 (118774.4-247447.3) | 309.3 (207.6-432.6) | -0.25 (-0.38 to -0.11) | **0** |
| 80-84 years | 46076.4 (30347.4-65178.3) | 349.5 (230.2-494.5) | 110938.4 (72722.3-158007.7) | 314.8 (206.4-448.4) | -0.32 (-0.49 to -0.15) | **0** |
| 85-89 years | 16146.5 (10924.5-22875.8) | 324.7 (219.7-460) | 48975.5 (33130.6-70017.6) | 300.8 (203.5-430) | -0.26 (-0.41 to -0.11) | **0.001** |
| 90-94 years | 3328.4 (2209.7-4855.8) | 267.8 (177.8-390.8) | 13543 (8970.2-19803.7) | 255.1 (169-373.1) | -0.14 (-0.24 to -0.04) | **0.008** |
| 95+ years | 561.4 (349.4-851.4) | 220.2 (137.1-334) | 2652.8 (1661.5-4020.6) | 207.8 (130.2-315) | -0.21 (-0.27 to -0.15) | **0** |
| Female |  |  |  |  |  |  |
| 65-69 years | 183304.6 (122466.5-259948.5) | 276.8 (184.9-392.5) | 349038 (230447.8-498359.3) | 258.6 (170.7-369.2) | -0.2 (-0.35 to -0.05) | **0.008** |
| 70-74 years | 153116.6 (103354.4-217927.5) | 325.8 (219.9-463.8) | 290652.5 (194614.3-416697.7) | 293.6 (196.6-421) | -0.32 (-0.46 to -0.17) | **0** |
| 75-79 years | 124688.4 (85612.5-175133.1) | 344 (236.2-483.1) | 225763.6 (153124.1-316906.4) | 323.2 (219.2-453.7) | -0.17 (-0.29 to -0.05) | **0.007** |
| 80-84 years | 76407.5 (50371-108004.7) | 346.7 (228.6-490.1) | 162319.1 (106446.9-229794.3) | 330 (216.4-467.2) | -0.14 (-0.3 to 0.02) | 0.086 |
| 85-89 years | 32277.6 (21746.7-46055.1) | 319.7 (215.4-456.2) | 83891.4 (56526-119467.7) | 308.5 (207.8-439.3) | -0.12 (-0.24 to -0.01) | **0.039** |
| 90-94 years | 8614.4 (5711.3-12601.1) | 272.3 (180.5-398.3) | 30266.7 (20220.2-44082.4) | 262.1 (175.1-381.7) | -0.12 (-0.2 to -0.04) | **0.002** |
| 95+ years | 1809.2 (1126.4-2735.4) | 233.6 (145.4-353.2) | 7687.2 (4827.3-11628.6) | 219.8 (138-332.5) | -0.2 (-0.23 to -0.17) | **0** |

YLDs, years lived with disability; AAPC, average annual percentage changes. p-values less than 0.05 are considered statistically significant and are highlighted in bold.
