# Supplementary material for: Trends in the global burden of vision loss among the older adults from 1990 to 2019
Source: Front Public Health. 2024 Apr 4;12:1324141. doi: 10.3389/fpubh.2024.1324141 (PMC11025641; doi:10.3389/fpubh.2024.1324141)
Supplement: Supplementary file 6 [file Data_Sheet_6.DOCX]

**Supplementary Table 6.** **Prevalence and Years Lived with Disability (YLDs) of Near Vision Loss and their average annual percentage changes (AAPCs) from 1990 to 2019 at the Global Level (Age>=65 Years)**

|  | Prevalence | | | |  |  |
| --- | --- | --- | --- | --- | --- | --- |
|  | case (n), 1990 | Prevalence (per 100,000 population), 1990 | case (n), 2019 | Prevalence (per 100,000 population), 2019 | AAPC, 1990-2019 | p value |
| Near vision loss |  |  |  |  |  |  |
| Male |  |  |  |  |  |  |
| 65-69 years | 14262921.2 (9116014.4-20532767.1) | 24910 (15921-35860.3) | 31376138.2 (20158547.3-45120689.7) | 25381.8 (16307.3-36500.5) | 0.06 (0.01 to 0.1) | **0.013** |
| 70-74 years | 10793303.3 (7053264.2-15366986.8) | 28765.6 (18797.9-40955) | 24527511.7 (16130512.5-34859552.9) | 27839.4 (18308.6-39566.5) | -0.12 (-0.14 to -0.09) | **0** |
| 75-79 years | 7597357.8 (5011803.9-11279934.3) | 30319 (20000.8-45015.2) | 17049260.8 (11205992-25620906.7) | 29803.8 (19589.2-44787.9) | -0.03 (-0.11 to 0.05) | 0.455 |
| 80-84 years | 4157688.1 (2729870.3-6184944) | 31541.4 (20709.6-46920.8) | 10861688.8 (7117271.4-16520094) | 30824 (20197.9-46881.8) | -0.05 (-0.17 to 0.06) | 0.368 |
| 85-89 years | 1553619.3 (1006891.8-2227697.5) | 31240.7 (20246.9-44795.3) | 4862407.9 (3142419.6-7183095.7) | 29861.3 (19298.4-44113.3) | -0.14 (-0.27 to -0.02) | **0.021** |
| 90-94 years | 365611.7 (226443.1-534658.7) | 29422.4 (18222.9-43026.3) | 1490441.6 (918312.4-2208342.8) | 28079.8 (17300.9-41605) | -0.15 (-0.27 to -0.02) | **0.024** |
| 95+ years | 71087.8 (39331.9-106417.5) | 27887.3 (15429.7-41747) | 330597.8 (177337.4-507300.5) | 25902 (13894.2-39746.5) | -0.23 (-0.39 to -0.07) | **0.005** |
| Female |  |  |  |  |  |  |
| 65-69 years | 17342043.2 (11064157.6-25009979.2) | 26184.2 (16705.4-37761.8) | 38144606.4 (24305841.1-54552994.5) | 28262.2 (18008.7-40419.5) | 0.26 (0.2 to 0.31) | **0** |
| 70-74 years | 14194072 (9319570-20138504.7) | 30205.4 (19832.3-42855.3) | 30463059.5 (19904786.9-43258611.1) | 30775.9 (20109.2-43702.9) | 0.04 (-0.02 to 0.11) | 0.186 |
| 75-79 years | 11501918 (7599845.4-16947247.1) | 31728.4 (20964.4-46749.5) | 23042958.5 (15245309.7-34398962.4) | 32989.8 (21826.2-49247.8) | 0.14 (0.02 to 0.26) | **0.02** |
| 80-84 years | 7088748.6 (4667606.7-10427083.1) | 32166 (21179.8-47314.1) | 16930863.1 (11109393.3-25061902.5) | 34423.1 (22587.2-50954.8) | 0.25 (0.14 to 0.36) | **0** |
| 85-89 years | 3146903.1 (2048274-4458936.9) | 31170.8 (20288.6-44166.8) | 8858385.5 (5729997.2-12866380.1) | 32570.5 (21068-47307) | 0.18 (0.04 to 0.32) | **0.011** |
| 90-94 years | 900080.6 (559088.3-1293398) | 28453.2 (17673.8-40886.6) | 3448825.6 (2131389.2-5081232.9) | 29860.7 (18454.1-43994.5) | 0.14 (0.01 to 0.26) | **0.03** |
| 95+ years | 207483 (115371.6-302171.8) | 26788.1 (14895.6-39013.3) | 883739.3 (482563.6-1339907) | 25272.3 (13799.9-38317.4) | -0.2 (-0.28 to -0.12) | **0** |
|  |  |  |  |  |  |  |
|  | YLDs | | | |  |  |
|  | case (n), 1990 | YLDs (per 100,000 population), 1990 | case (n), 2019 | YLDs (per 100,000 population), 2019 | AAPC, 1990-2019 | p value |
| Near vision loss |  |  |  |  |  |  |
| Male |  |  |  |  |  |  |
| 65-69 years | 141149.2 (60292.3-283872.5) | 246.5 (105.3-495.8) | 311436.2 (132750.7-627094.5) | 251.9 (107.4-507.3) | 0.07 (0.02 to 0.12) | **0.004** |
| 70-74 years | 105218 (44823.2-208971.5) | 280.4 (119.5-556.9) | 239668.6 (101711.8-472167.2) | 272 (115.4-535.9) | -0.11 (-0.13 to -0.08) | **0** |
| 75-79 years | 72932 (31123-147271.3) | 291.1 (124.2-587.7) | 164000.3 (69677-328567.9) | 286.7 (121.8-574.4) | -0.02 (-0.11 to 0.07) | 0.643 |
| 80-84 years | 39296.7 (16878.4-77667.8) | 298.1 (128-589.2) | 102911.6 (44264.3-202700.7) | 292 (125.6-575.2) | -0.04 (-0.16 to 0.08) | 0.485 |
| 85-89 years | 14476.5 (6370.9-27355.9) | 291.1 (128.1-550.1) | 45372.7 (19688.4-85746.2) | 278.6 (120.9-526.6) | -0.14 (-0.27 to -0.01) | **0.03** |
| 90-94 years | 3365.3 (1445.7-6420.1) | 270.8 (116.3-516.7) | 13738.3 (5864.8-26269.1) | 258.8 (110.5-494.9) | -0.14 (-0.27 to -0.01) | **0.03** |
| 95+ years | 643.4 (259.6-1311.8) | 252.4 (101.8-514.6) | 3001.7 (1178.7-6218.5) | 235.2 (92.3-487.2) | -0.22 (-0.37 to -0.07) | **0.004** |
| Female |  |  |  |  |  |  |
| 65-69 years | 170429.2 (71561.4-345639) | 257.3 (108-521.9) | 375584.3 (159962.8-752580) | 278.3 (118.5-557.6) | 0.26 (0.2 to 0.31) | **0** |
| 70-74 years | 137616.4 (59103.8-273528.1) | 292.9 (125.8-582.1) | 295831.4 (127231.4-583636.3) | 298.9 (128.5-589.6) | 0.05 (-0.01 to 0.11) | 0.105 |
| 75-79 years | 109973.5 (46758.5-221981.6) | 303.4 (129-612.3) | 220371.4 (93760-443482.9) | 315.5 (134.2-634.9) | 0.14 (0.02 to 0.26) | **0.02** |
| 80-84 years | 66650.6 (28677.7-132550.6) | 302.4 (130.1-601.5) | 159263 (68152-318773.6) | 323.8 (138.6-648.1) | 0.25 (0.13 to 0.37) | **0** |
| 85-89 years | 29133.5 (12721.1-54639.1) | 288.6 (126-541.2) | 81943.3 (36465.8-152955.3) | 301.3 (134.1-562.4) | 0.18 (0.03 to 0.33) | **0.017** |
| 90-94 years | 8210.2 (3522.7-15408.6) | 259.5 (111.4-487.1) | 31425.6 (13506.4-59818.9) | 272.1 (116.9-517.9) | 0.13 (0 to 0.26) | **0.045** |
| 95+ years | 1858.2 (758.3-3726.5) | 239.9 (97.9-481.1) | 7898.4 (3172.1-16326) | 225.9 (90.7-466.9) | -0.2 (-0.28 to -0.12) | **0** |

YLDs, years lived with disability; AAPC, average annual percentage changes. p-values less than 0.05 are considered statistically significant and are highlighted in bold.
