# Supplementary material for: Trends in the global burden of vision loss among the older adults from 1990 to 2019
Source: Front Public Health. 2024 Apr 4;12:1324141. doi: 10.3389/fpubh.2024.1324141 (PMC11025641; doi:10.3389/fpubh.2024.1324141)
Supplement: Supplementary file 7 [file Data_Sheet_7.DOCX]

**Supplementary Table 7.** **Prevalence and Years Lived with Disability (YLDs) of Other Causes and their average annual percentage changes (AAPCs) from 1990 to 2019 at the Global Level (Age>=65 Years)**

|  | Prevalence | | | |  |  |
| --- | --- | --- | --- | --- | --- | --- |
|  | case (n), 1990 | Prevalence (per 100,000 population), 1990 | case (n), 2019 | Prevalence (per 100,000 population), 2019 | AAPC, 1990-2019 | p value |
| Other causes |  |  |  |  |  |  |
| Male |  |  |  |  |  |  |
| 65-69 years | 1236466 (987056.4-1535515.2) | 2159.5 (1723.9-2681.8) | 986225.3 (783495-1252192.2) | 2159.7 (1727.3-2666.2) | -0.01 (-0.07 to 0.06) | 0.834 |
| 70-74 years | 958781.1 (778419.8-1190117.1) | 2555.3 (2074.6-3171.8) | 555726.6 (427387.7-706141.2) | 2466.7 (2009.2-3034.3) | -0.1 (-0.18 to -0.02) | **0.015** |
| 75-79 years | 656999.7 (515286.4-832417.8) | 2621.9 (2056.4-3322) | 326949.2 (259967.3-406035.6) | 2472.9 (1946.5-3109.6) | -0.18 (-0.34 to -0.02) | **0.028** |
| 80-84 years | 402410.8 (319874.5-515298.6) | 3052.8 (2426.7-3909.2) | 114982 (87450.4-150443.1) | 2798.8 (2223.5-3553.6) | -0.28 (-0.3 to -0.25) | **0** |
| 85-89 years | 193605.2 (148554.2-246740.3) | 3893.1 (2987.2-4961.5) | 2669774 (2135258.2-3295829.8) | 3412.9 (2624.7-4336.6) | -0.47 (-0.6 to -0.34) | **0** |
| 90-94 years | 81059.4 (64387.8-100671.9) | 6523.2 (5181.6-8101.5) | 2173261.3 (1770189.9-2673304.4) | 6159.7 (4897.8-7649.7) | -0.18 (-0.31 to -0.06) | **0.003** |
| 95+ years | 23945.3 (18107-31447.3) | 9393.6 (7103.3-12336.6) | 1414611.7 (1113507.8-1778875) | 9008.7 (6851.6-11787.1) | -0.14 (-0.24 to -0.04) | **0.006** |
| Female |  |  |  |  |  |  |
| 65-69 years | 1648985.8 (1303942-2051006.5) | 2489.8 (1968.8-3096.8) | 3325433.5 (2634444.2-4144576.2) | 2463.9 (1951.9-3070.8) | -0.05 (-0.15 to 0.06) | 0.403 |
| 70-74 years | 1391041.8 (1124441-1728621.9) | 2960.2 (2392.8-3678.6) | 2814341.5 (2279046.5-3498551.8) | 2843.2 (2302.5-3534.5) | -0.17 (-0.39 to 0.04) | 0.118 |
| 75-79 years | 1137220.7 (879965.7-1449152) | 3137.1 (2427.4-3997.5) | 2050603.4 (1600659.1-2595336.3) | 2935.8 (2291.6-3715.7) | -0.16 (-0.32 to 0.01) | 0.06 |
| 80-84 years | 794132.9 (624949.6-1012722.8) | 3603.5 (2835.8-4595.3) | 1667106.3 (1322091-2126043.1) | 3389.5 (2688-4322.6) | -0.25 (-0.39 to -0.11) | **0** |
| 85-89 years | 455948.2 (353449.1-579727.9) | 4516.3 (3501-5742.3) | 1068637.9 (826781.5-1358076.8) | 3929.2 (3039.9-4993.4) | -0.47 (-0.55 to -0.4) | **0** |
| 90-94 years | 206762.2 (164442-257154.3) | 6536.1 (5198.3-8129.1) | 706723.2 (565942.1-878857.8) | 6119 (4900.1-7609.4) | -0.25 (-0.34 to -0.16) | **0** |
| 95+ years | 70605.9 (54373.1-91898.2) | 9115.9 (7020.1-11864.9) | 274960.5 (210156.1-363071.9) | 7863.1 (6009.8-10382.8) | -0.54 (-0.66 to -0.42) | **0** |
|  |  |  |  |  |  |  |
|  | YLDs | | | |  |  |
|  | case (n), 1990 | YLDs (per 100,000 population), 1990 | case (n), 2019 | YLDs (per 100,000 population), 2019 | AAPC, 1990-2019 | p value |
| Other causes |  |  |  |  |  |  |
| Male |  |  |  |  |  |  |
| 65-69 years | 100660.8 (68678.6-142008.3) | 175.8 (119.9-248) | 201129.2 (136045.9-284680.2) | 162.7 (110.1-230.3) | -0.26 (-0.32 to -0.21) | **0** |
| 70-74 years | 80005.9 (55563.3-114218.8) | 213.2 (148.1-304.4) | 169051.4 (117245-240203.4) | 191.9 (133.1-272.6) | -0.33 (-0.41 to -0.25) | **0** |
| 75-79 years | 55907.1 (38044.6-80483.5) | 223.1 (151.8-321.2) | 115817.4 (79011.7-165861.7) | 202.5 (138.1-289.9) | -0.35 (-0.5 to -0.2) | **0** |
| 80-84 years | 33463 (22807.6-47283.4) | 253.9 (173-358.7) | 79268 (54271.4-110672.3) | 225 (154-314.1) | -0.39 (-0.5 to -0.28) | **0** |
| 85-89 years | 15420.9 (10408.5-22143.3) | 310.1 (209.3-445.3) | 42496.2 (28906.2-60394.9) | 261 (177.5-370.9) | -0.58 (-0.67 to -0.48) | **0** |
| 90-94 years | 6348.7 (4415.7-8899.3) | 510.9 (355.4-716.2) | 24089.5 (16926.3-33093.4) | 453.8 (318.9-623.5) | -0.45 (-0.6 to -0.3) | **0** |
| 95+ years | 1872.3 (1222-2686.6) | 734.5 (479.4-1054) | 8439.5 (5596-11856.7) | 661.2 (438.4-929) | -0.38 (-0.47 to -0.28) | **0** |
| Female |  |  |  |  |  |  |
| 65-69 years | 118703.9 (80801.4-167097) | 179.2 (122-252.3) | 229468.8 (155569.2-323435.4) | 170 (115.3-239.6) | -0.19 (-0.25 to -0.13) | **0** |
| 70-74 years | 103921.7 (72097.1-147278.2) | 221.1 (153.4-313.4) | 203962 (141832.1-289053.9) | 206.1 (143.3-292) | -0.26 (-0.39 to -0.14) | **0** |
| 75-79 years | 86371.8 (58738.5-123108.8) | 238.3 (162-339.6) | 156210.3 (106794.1-221966.6) | 223.6 (152.9-317.8) | -0.22 (-0.39 to -0.04) | **0.017** |
| 80-84 years | 60057 (40968-83017.6) | 272.5 (185.9-376.7) | 124587.5 (85298-171933.8) | 253.3 (173.4-349.6) | -0.25 (-0.47 to -0.03) | **0.029** |
| 85-89 years | 33437.8 (22728.7-47215.4) | 331.2 (225.1-467.7) | 77274.5 (52777.7-108693.6) | 284.1 (194.1-399.6) | -0.51 (-0.71 to -0.32) | **0** |
| 90-94 years | 15774.9 (10987.4-21693.4) | 498.7 (347.3-685.8) | 52957 (37205.3-71894) | 458.5 (322.1-622.5) | -0.3 (-0.44 to -0.16) | **0** |
| 95+ years | 5701.5 (3839.4-7990.5) | 736.1 (495.7-1031.6) | 21383.9 (14346.6-30023.5) | 611.5 (410.3-858.6) | -0.67 (-0.79 to -0.56) | **0** |

YLDs, years lived with disability; AAPC, average annual percentage changes. p-values less than 0.05 are considered statistically significant and are highlighted in bold.
