# Supplementary material for: Trends in the global burden of vision loss among the older adults from 1990 to 2019
Source: Front Public Health. 2024 Apr 4;12:1324141. doi: 10.3389/fpubh.2024.1324141 (PMC11025641; doi:10.3389/fpubh.2024.1324141)
Supplement: Supplementary file 8 [file Data_Sheet_8.DOCX]

Supplementary Material

##

Supplementary Figures

**Figure S1.** Changes in specific types of vision loss YLDs according to population-level determinants of population growth, aging and epidemiological change from 1990 to 2019 at the global level and by SDI quintile. YLDs, years lived with disability, SDI, socio-demographic index.

**
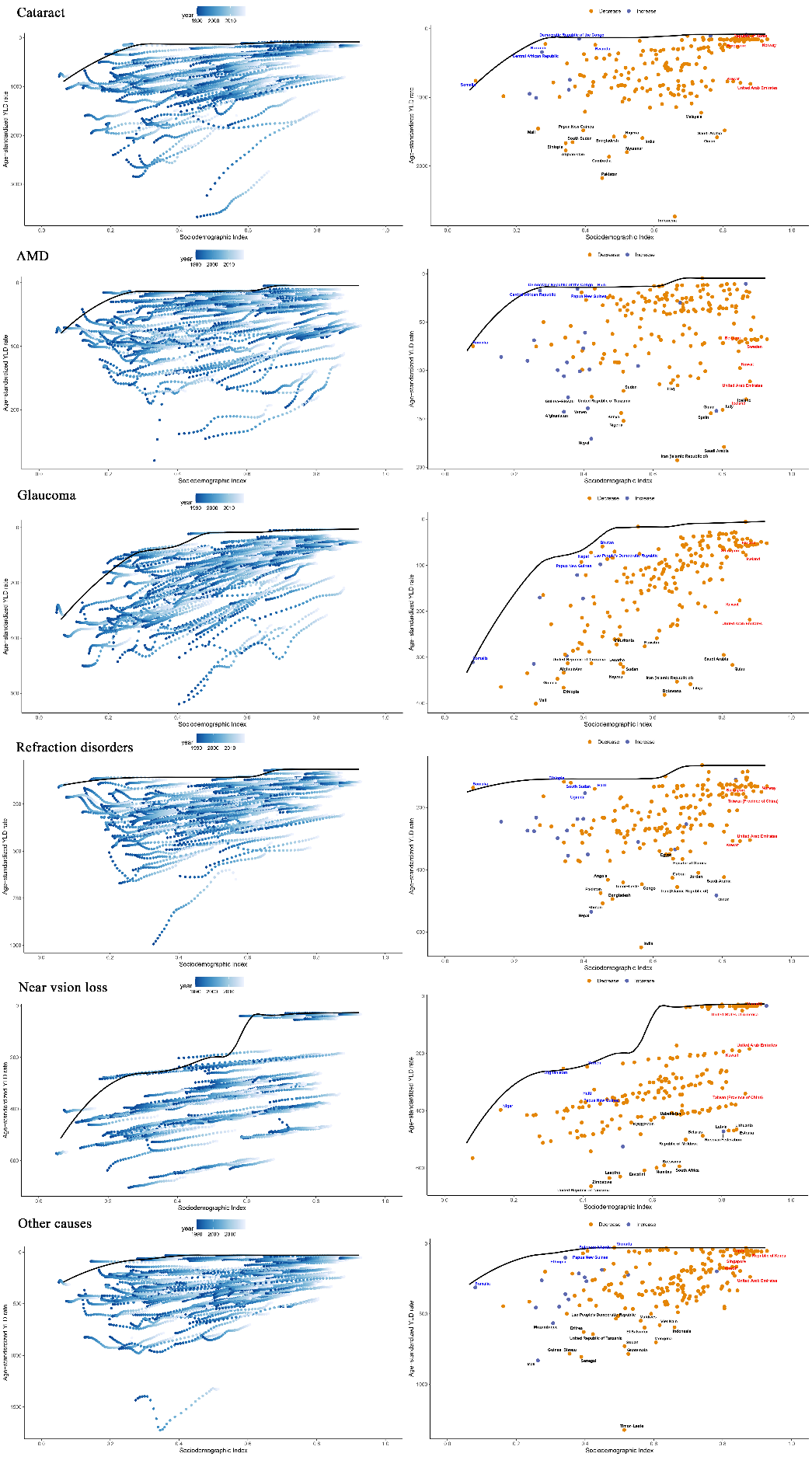
Figure S2.** Frontier analysis based on SDI and age-standardized specific types of vision loss (glaucoma, cataracts, AMD, refractive disorders, near vision loss and other causes) YLDs rate from 1990 to 2019 (left column). Frontier analysis based on SDI and age-standardized overall vision loss YLDs rate in 2019 (right column).
